# Supplementary material for: The global phosphorylation landscape of mouse oocytes during meiotic maturation
Source: EMBO J. 2024 Sep 10;43(20):4752–85. doi: 10.1038/s44318-024-00222-1 (PMC11480333; doi:10.1038/s44318-024-00222-1)
Supplement: Supplementary file 1 — Appendix [file 44318_2024_222_MOESM1_ESM.pdf]

Table of content of the Appendix Figures

The global phosphorylation landscape of mouse oocytes during meiotic maturation

Hongzheng Sun, Longsen Han, Yueshuai Guo, Huiqing An, Bing Wang, Xiangzheng Zhang, Jiashuo Li, Yingtong Jiang, Yue Wang, Guangyi Sun, Shuai Zhu, Shoubin Tang, Juan Ge, Minjian Chen, Xuejiang Guo, Qiang Wang

Table of Contents

Appendix Figure S1 Page 2

Appendix Figure S2 Page 3

Appendix Figure S3 Page 4

Appendix Figure S4 Page 5

Appendix Figure S5 Page 6

Appendix Figure S6 Page 7

Appendix Figure S7 Page 8

Appendix Figure S8 Page 9

Appendix Figure S9 Page 10

Appendix Figure S1

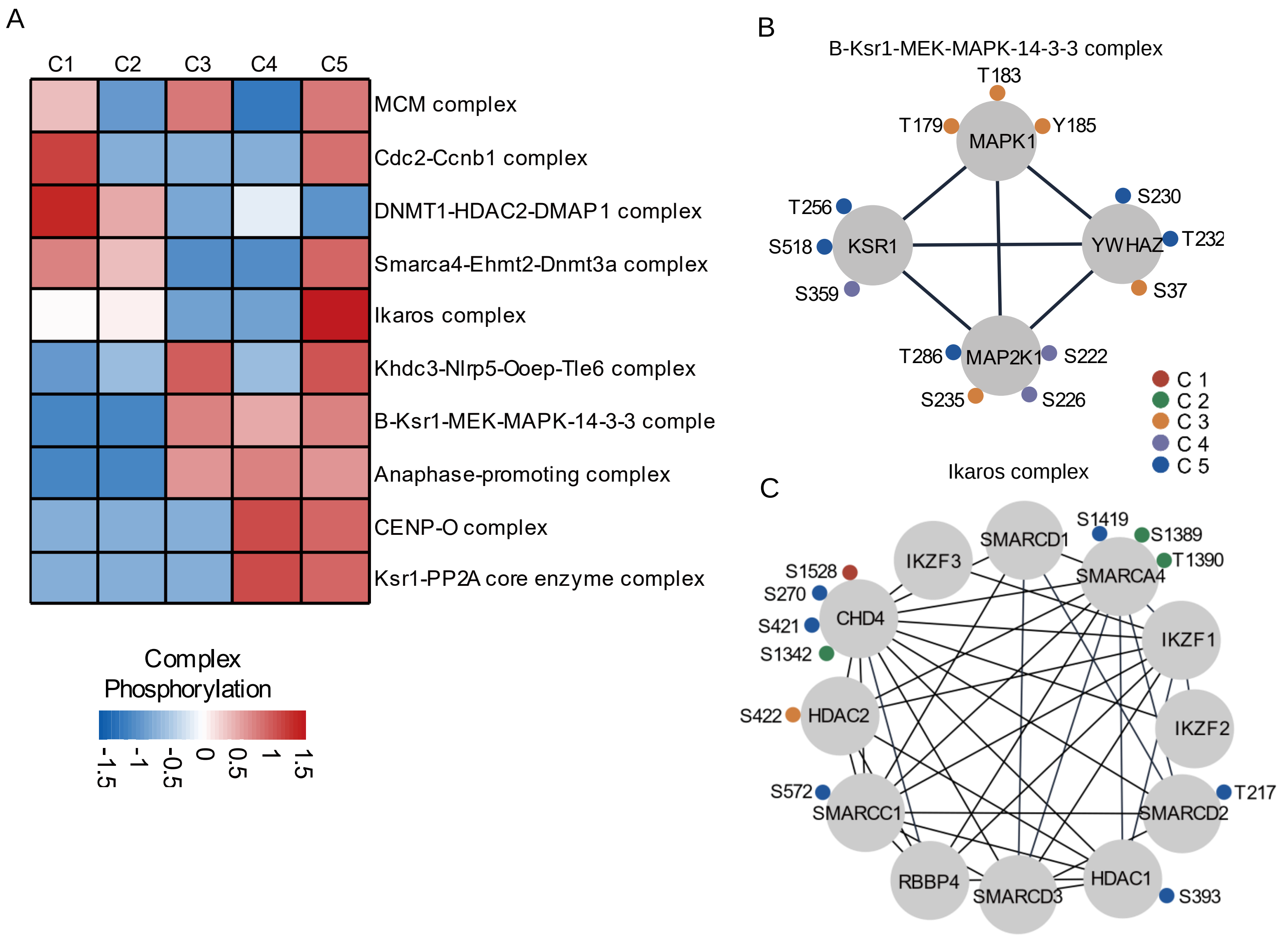

Appendix Figure S1. Protein complexes with regulated phosphosites. Related to Figure 2.
A. Overall phosphorylation change (log10(p)) in a protein complex, based on the phosphorylation change in member proteins. Only proteins with a significant change in phosphorylation at one site were included for calculating.
B-C. Representative complexes displaying proteins and phosphosites in networks. Functional association network generated using STRING and visualized with Cytoscape.

Appendix Figure S2

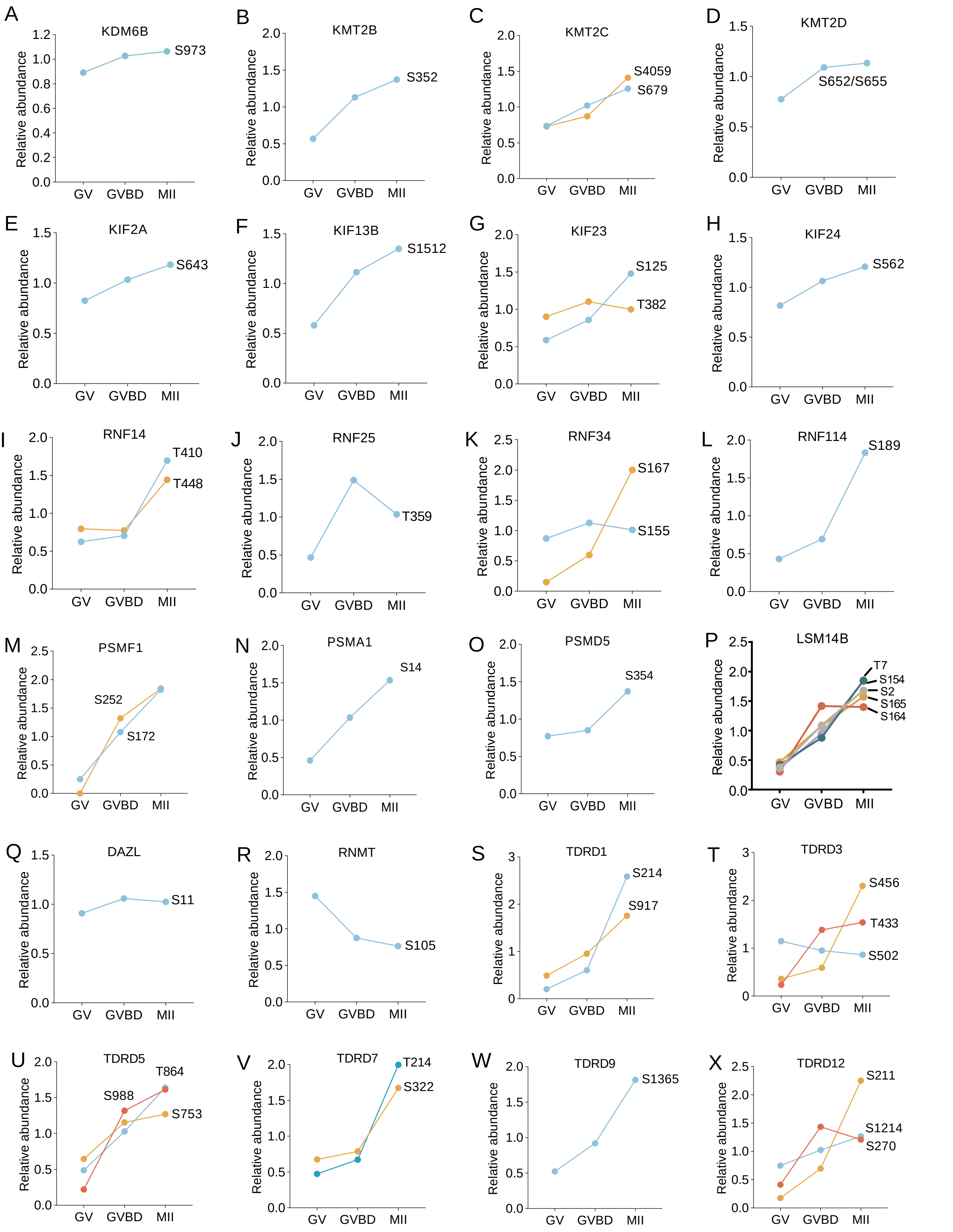

Appendix Figure S2. Dynamic changes of novel phosphosites during oocyte maturation. Related to Figure 3. A-X. Abundance profiles of novel phosphosites on the representative proteins in oocytes at GV, GVBD, and MII stages. Data are expressed as average value from five independent replicates.

Appendix Figure S3

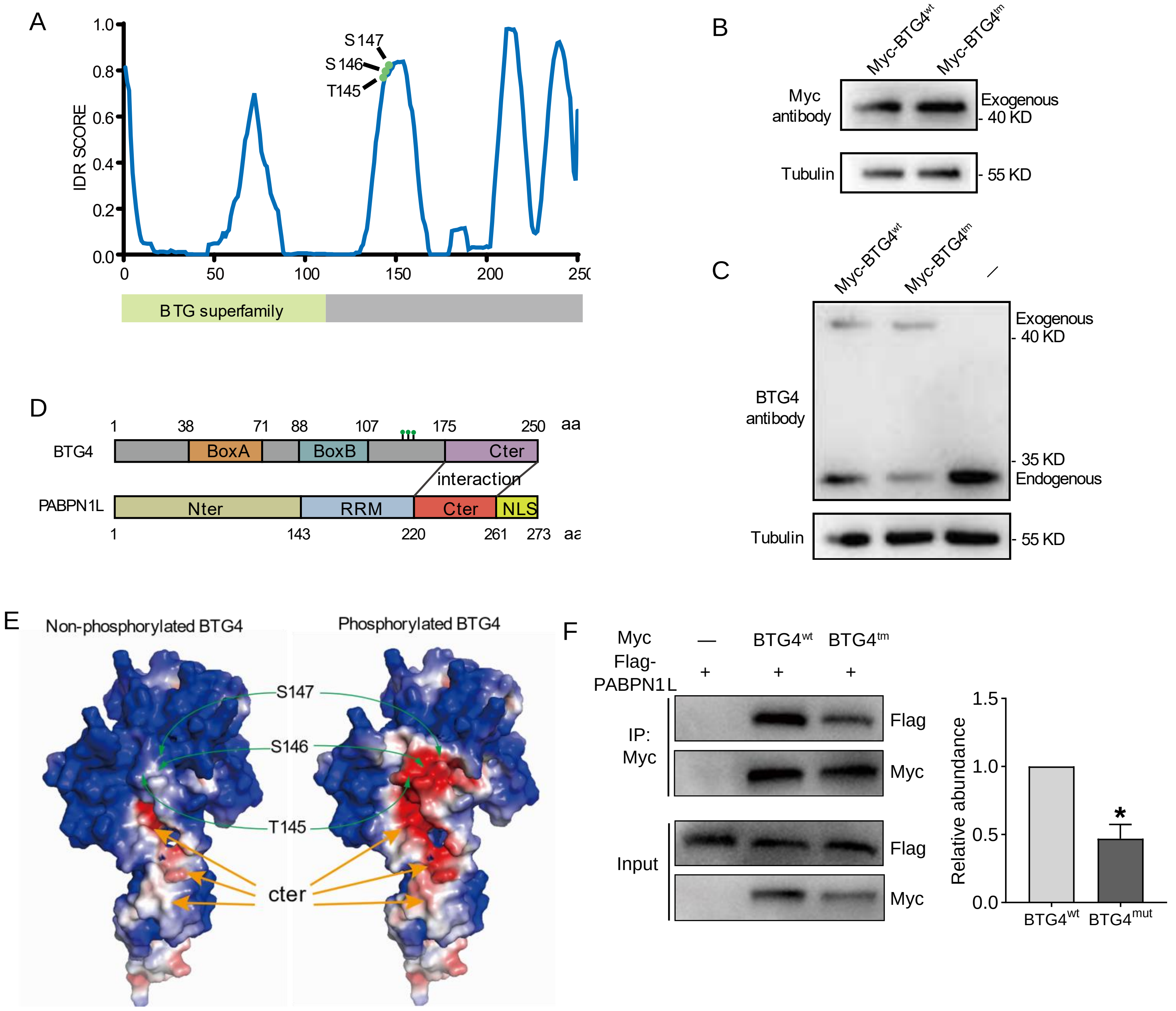

Appendix Figure S3. Functional evaluation of BTG4 phosphorylation in oocytes. Related to Figure 4.

A. A diagram showing mouse BTG4 with disordered regions predicted by IUPred2A online tool. Three phosphosites (T145, S146, and S147) are highlighted by green dots. IDR, intrinsically disordered region.

B-C. Immunoblotting showing the overexpression of wild-type BTG4 ($BTG4^{wt}$) and phosphomutant BTG4 ($BTG4^{tm}$) protein in oocytes. (100 oocytes per lane).

D. Diagrams of mouse BTG4 and PABPN1L constructs. Three phosphosites (T145, S146, and S147) are highlighted by green dots.

E. Surface electrostatic potential of non-phosphorylated (left) and phosphorylated (right) BTG4 protein. Positions of three phosphosites are indicated by green arrows. C-terminal is indicated by orange arrows. Electrostatic potential was computed with the Advanced Poisson-Boltzmann Solver (APBS) tool after preparation with the PDB2PQR tool.

F. Co-IP experiment showing the interactions between PABPN1L and BTG4. Lysates from 500 oocytes expressing MYC-BTG4 (wild-type or mutants shown in (B and C)) and FLAG-PABPN1L were immunoprecipitated with an anti-Myc antibody. The immunoprecipitated proteins are detected by Western blot with the indicated antibodies (500 oocytes for per Co-IP experiment). Welch's T-test was used for statistical analysis (n=3). Error bars, SD. * $p<0.05$.

Appendix Figure S4

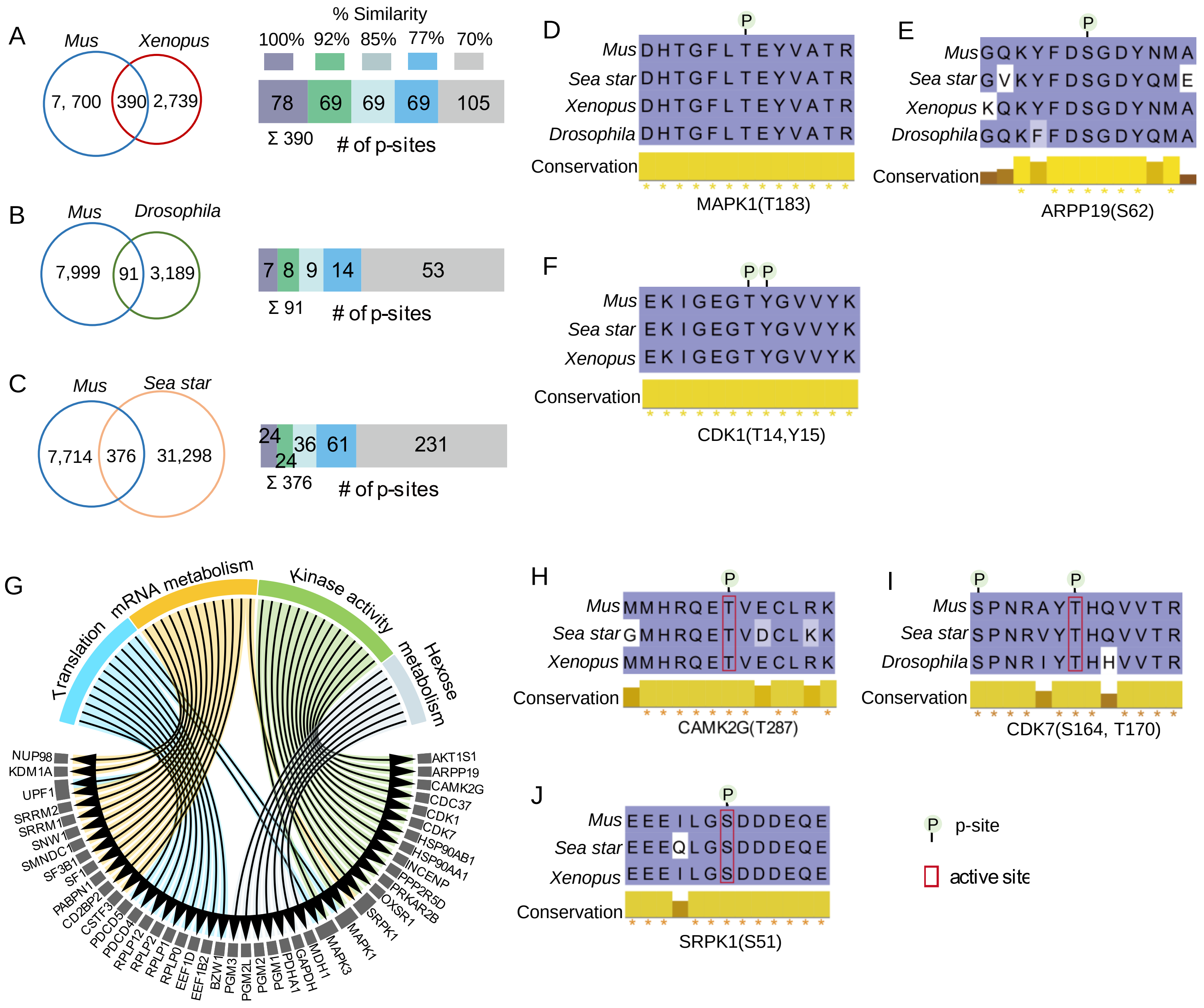

Appendix Figure S4. Conservation analysis of oocyte phosphosites. Related to Figure 5.

A-C. Comparative analysis of phosphoproteomes between mouse oocytes and oocytes from *Xenopus* (A), or *Drosophila* (B), or *starfish* (C).

D-F. Alignment of MAPK1 (D), ARPP19 (E), and CDK1 (F) sequences surrounding conserved phosphosites by Jalview Multiple Alignment.

G. GOChord plot of representative GO terms enriched for proteins with conserved phosphosites (p value < 0.05).

H-J. Alignment of CAMK2G (H), CDK7 (E), and SRPK1 (F) sequences surrounding conserved phosphosites.

Appendix Figure S5

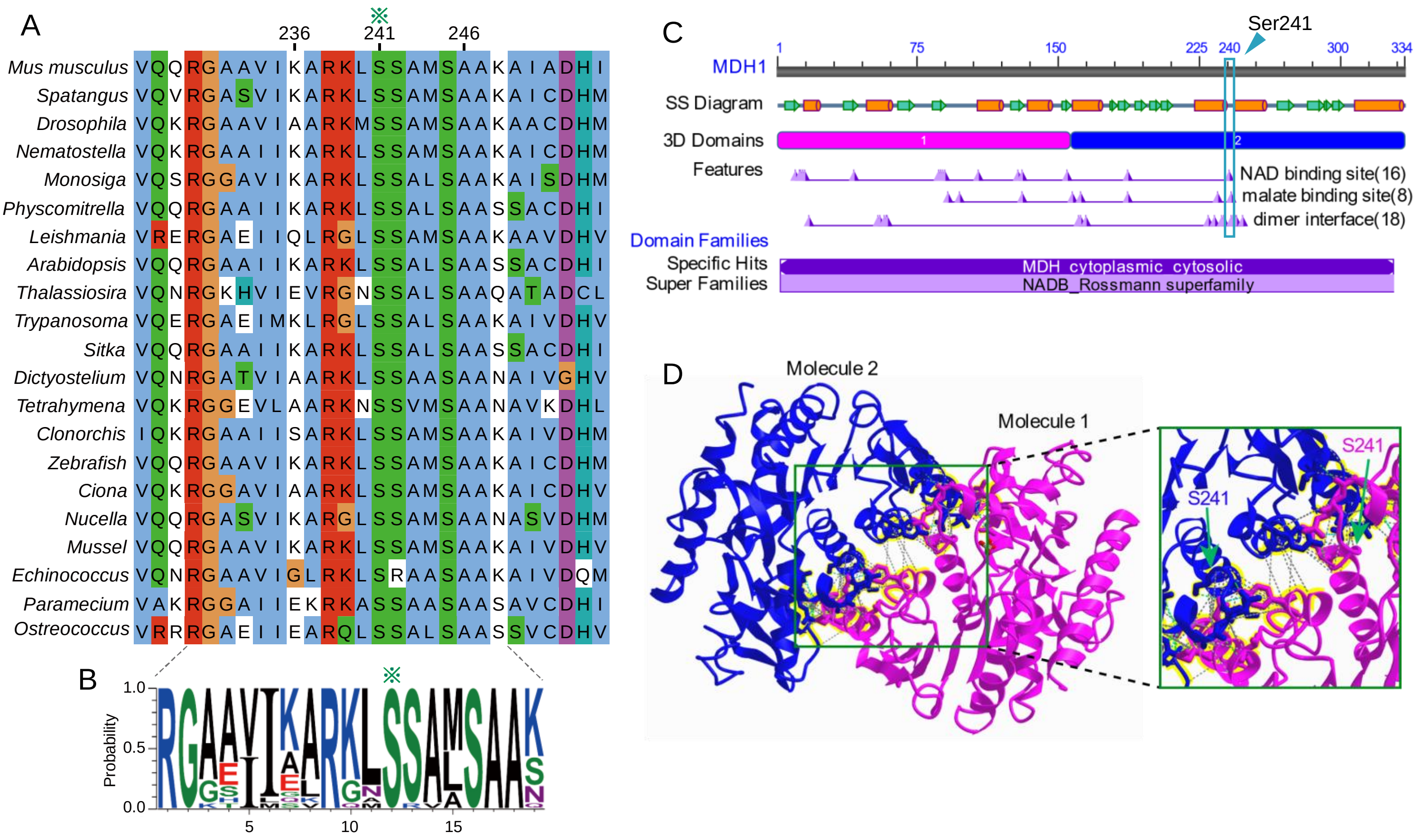

Appendix Figure S5. Conservation analysis and molecular feature of MDH1. Related to Figure 5.

A. Multiple alignments of MDH1 sequence surrounding Ser241 (mouse).

B. Sequence logo of 19 amino acids flanking conserved phosphosites of MDH1.

C. Schematic diagram of MDH1 protein domain structures with functional motifs/sites.

D. The three-dimensional structures for MDH1 protein dimer (PDB: 7RM9).

Appendix Figure S6

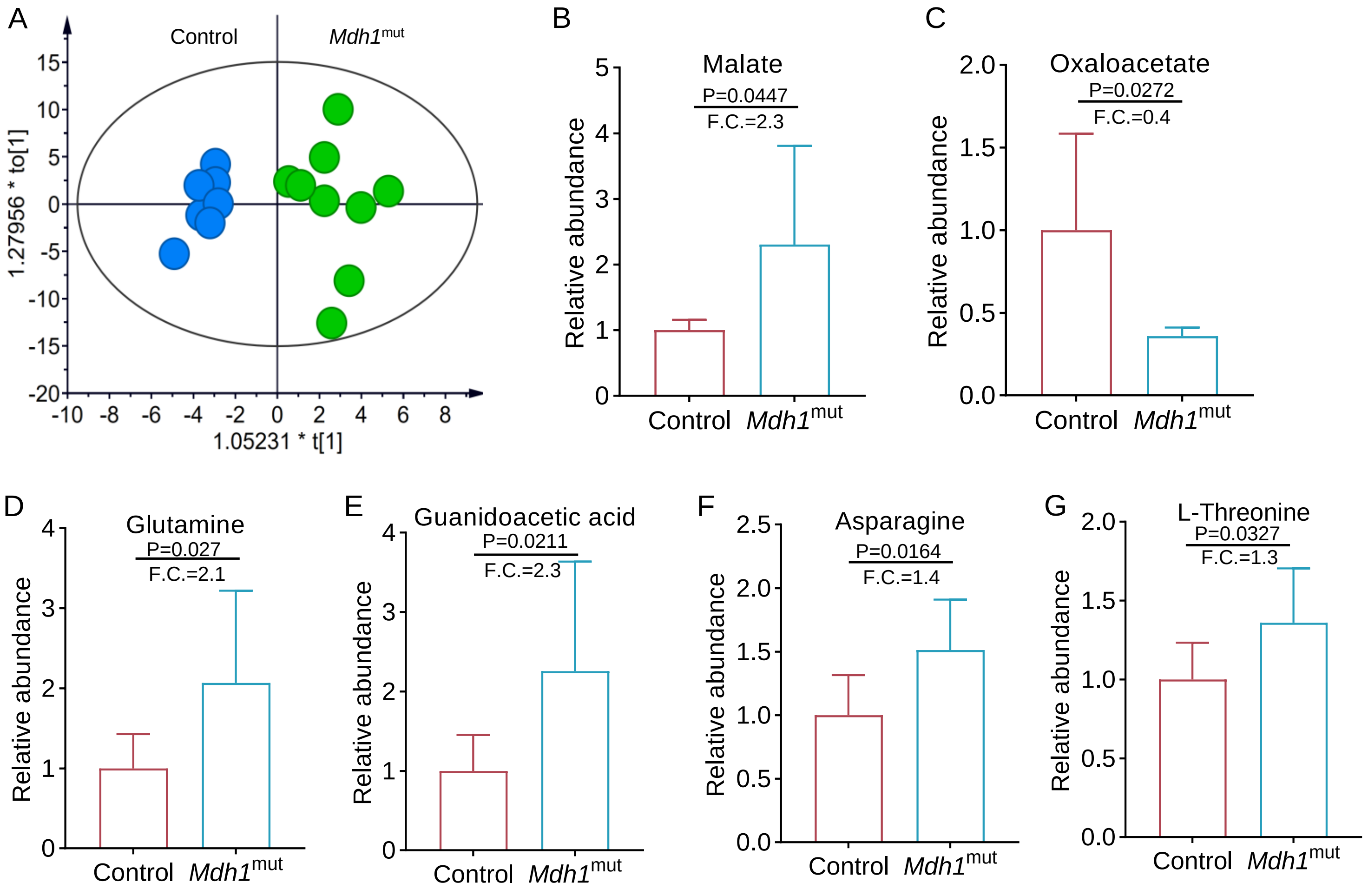

Appendix Figure S6. Metabolic changes in oocytes with MDH1 phosphomutant. Related to Figure 5.

A. OPLS-DA score plot separating control oocyte and *Mdh1*^{mut} oocyte samples.

B-C. Bar chart showing the relative level of malate (B) and oxaloacetate (C) in control group and *Mdh1*^{mut} group.

D-E. Bar chart showing the relative level of different amino acids in control group and *Mdh1*^{mut} group.

For statistical analysis, Welch's T-test was applied to sections B-E, while a two-tailed Student's t-test was utilized for sections F and G. Error bars, SD. Control group, n = 7 replicates; *Mdh1*^{mut}, n = 9 replicates.

Appendix Figure S7

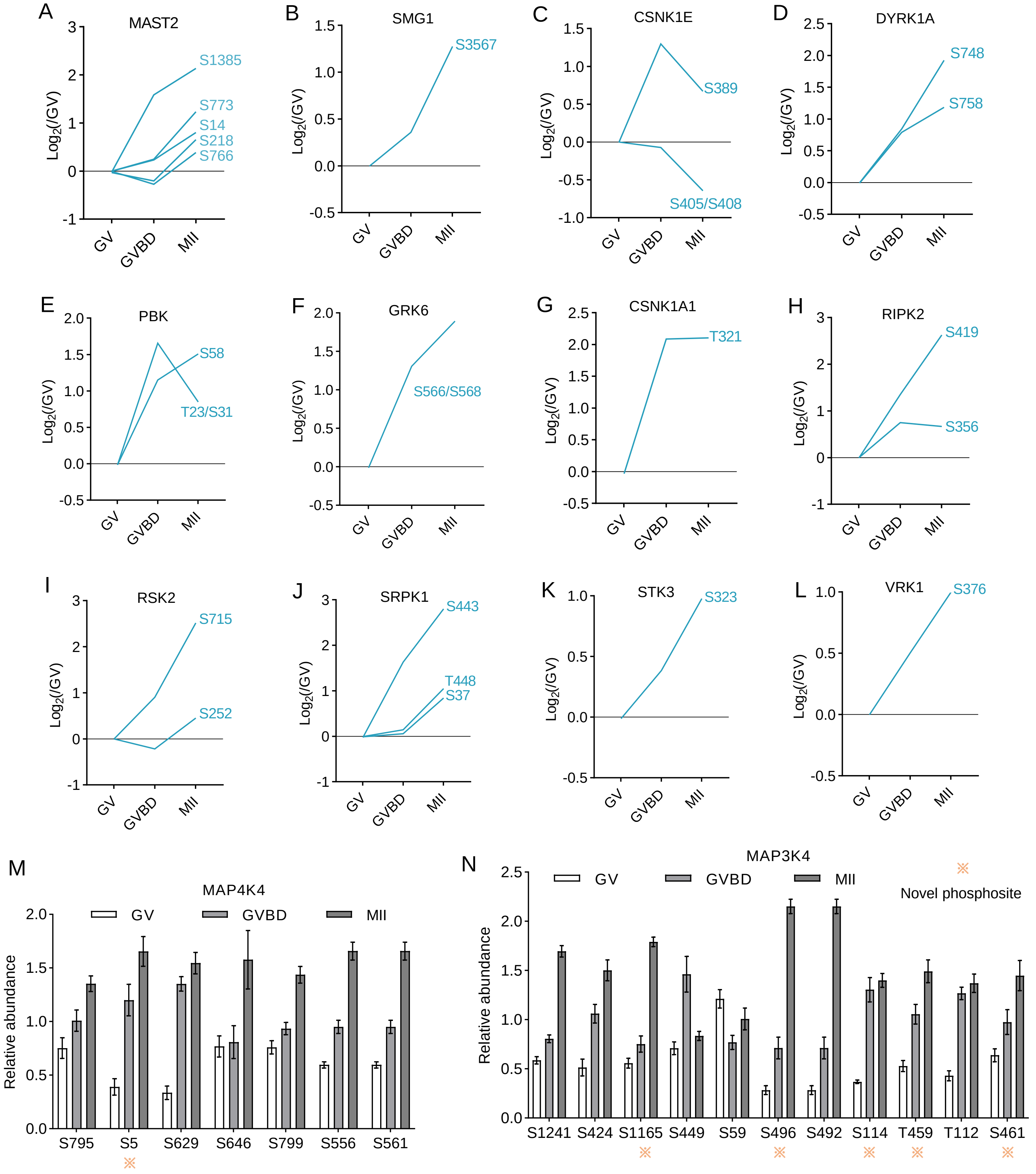

Appendix Figure S7. Phosphorylation dynamics of the representative kinases in oocytes. Related to Figure 6.

A-L. Regulated phosphosites on 12 representative kinases.

M-N. Bar chart showing the relative levels of regulated phosphosites on MAP4K4 (M) and MAP3K4 (N). The novel phosphosites were donated with asterisks. Data are expressed as mean percentage ±SD from five independent replicates.

Appendix Figure S8

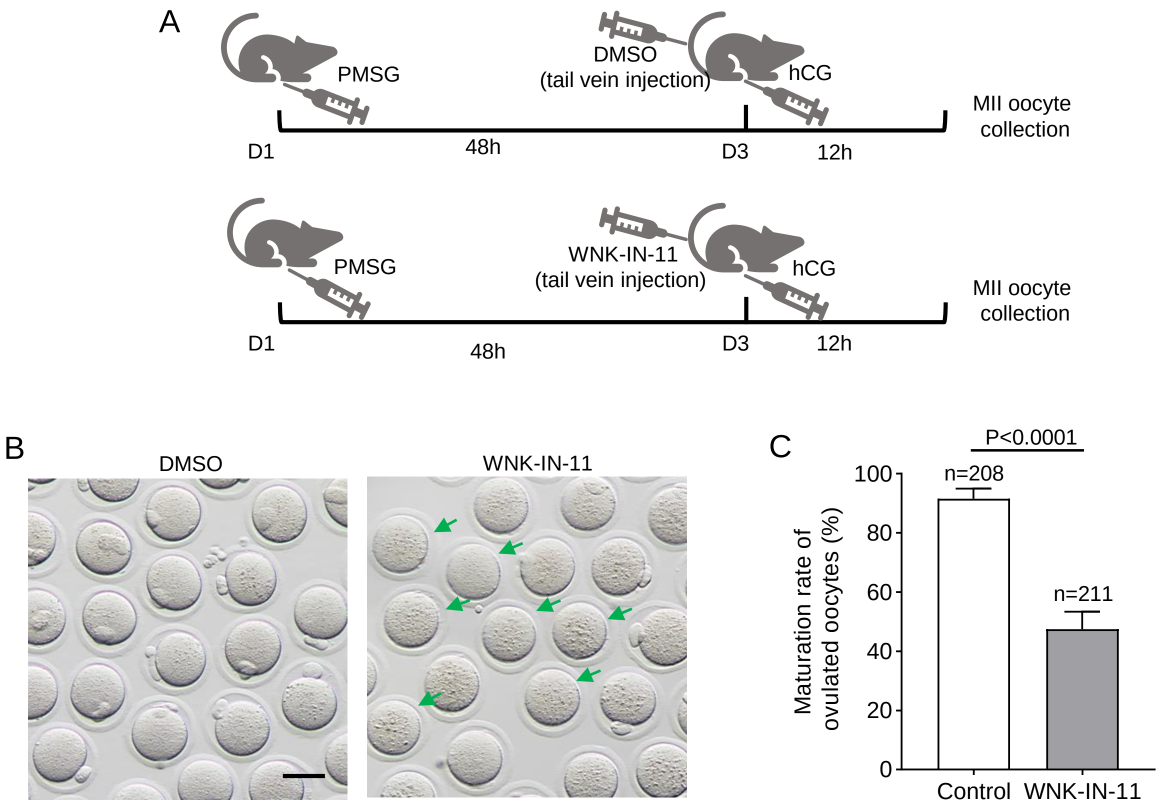

Appendix Figure S8. Effect of *in vivo* administration of WNK1 inhibitor on oocyte development. Related to Figure 6.

A. Schematic diagram illustrating the experimental procedure for *in vivo* treatment with WNK-IN-11.

B. Bright-field images of oocytes collected from DMSO-treated mice (left) and WNK-IN-11-treated mice (right). Arrowheads point to oocytes that fail to extrude a polar body. Scale bars, 50 μm.

C. Quantitative analysis of ovulated oocytes with polar body. Experiments were conducted with 6 mice in DMSO-treatment group and 10 mice in WNK-IN-11 treatment group. Data are expressed as mean percentage ± SD (Control: *n*=6 replicates, 208 oocytes; WNK-IN-1: *n*=10 replicates, 211 oocytes). Two-tailed Student's t test was used for statistical analysis, comparing to control group (DMSO treatment).

Appendix Figure S9

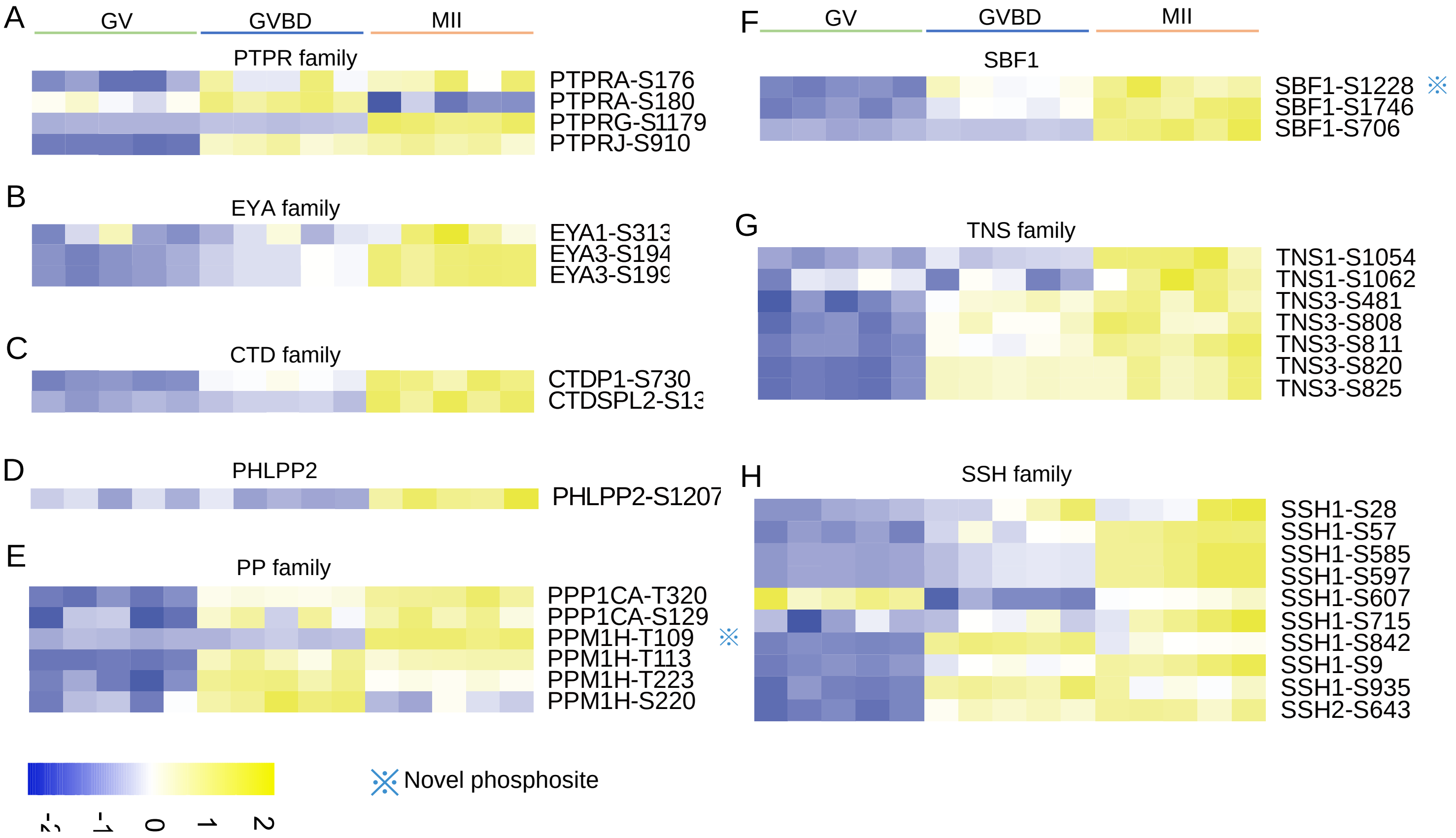

Appendix Figure S9. Phosphorylation dynamics on phosphatases during meiotic maturation. Related to Figure. 7.

A-H. Heatmap showing the dynamic phosphorylation of phosphatases grouped into distinct families. Novel phosphosites identified within each phosphatase are marked with blue asterisks.
