# Supplementary material for: The global phosphorylation landscape of mouse oocytes during meiotic maturation
Source: EMBO J. 2024 Sep 10;43(20):4752–85. doi: 10.1038/s44318-024-00222-1 (PMC11480333; doi:10.1038/s44318-024-00222-1)
Supplement: Supplementary file 2 — Table EV1 [file 44318_2024_222_MOESM2_ESM.docx]

| Primer sequences for overexpression analysis |
| --- |
| Gene Primer sequence |
| Btg4 F: CAGGCCGGCCCATGAGAGACGAAATTGCAACA  R: GAGGCGCGCCCTCATTTTTGCTTTAGGGAAGA  Btg4 F: TGGCGCAGCTGCTGATGAAGAAAGCTGCAGCAGGG  (mutant) R: TCAGCAGCTGCGCCAGAGGAGCAGTCTCCC    Pabpn1l F: GGAATTCAATGGAGCCTTACCTGAGCA  R: GCTCTAGAGGCTGTTCTTTCAGTAGGGA  Mdh1 F: GGAATTCAATGTCTGAACCAATCAGAGT  R: GCTCTAGATCACGCAGAGGAGAGAAACT  Mdh1 F: GAAGCTGGCCAGTGCAATGTCTGCTGCGA  (mutant) R: GCACTGGCCAGCTTCCGAGCCTTGATGACA  Rpl12 F: GGAATTCCATGCCGCCCAAGTTCGACC  R: GCTCTAGATTAACTAGCTGGGCACTCCACTGC  Rpl12 F: GGGTCTGGCTCCGAAGAAAGTTGGCGATGAC  (mutant) R: TTCGGAGCCAGACCCAGAGGACCGATC |

Primer sequences for Poly(A) tail (PAT) assay

| Gene Primer sequence |
| --- |
| Anchor:P1 5’ –P–GGTCACCTTGATCTGAAGC–NH2– 3’  Anti-anchor:P2 5’ –GCTTCAGATCAAGGTGACCTTTTT– 3’  Zp3 5’-ACCGCAGGCACGTGAC-3’  Gtsf1 5’-GTGGCAACAACAGTCCTGC-3’  Gapdh 5’- ACTGAGCAAGAGAGGCCCTA-3’ |

Primer sequences for quantitative real-time PCR

| Gene Primer sequence |
| --- |
| Zp3 F: 5’- ATGGCGTCAAGCTATTTCCTC -3’  R: 5’- CGTGCCAAAAAGGTCTCTACT -3’  Gtsf1 F: 5’- AAATTGGCTACTTGTCCCTTCAA -3’  R: 5’- TCCTGCTCAATACAGCTCTTGT -3’  Gapdh F: 5’- CTTTGTCAAGCTCATTTCCTGG -3’  R: 5’- TCTTGCTCAGTGTCCTTGC- 3’ |
