# Supplementary figures and images for: The global phosphorylation landscape of mouse oocytes during meiotic maturation
Source: EMBO J. 2024 Sep 10;43(20):4752–85. doi: 10.1038/s44318-024-00222-1 (PMC11480333; doi:10.1038/s44318-024-00222-1)

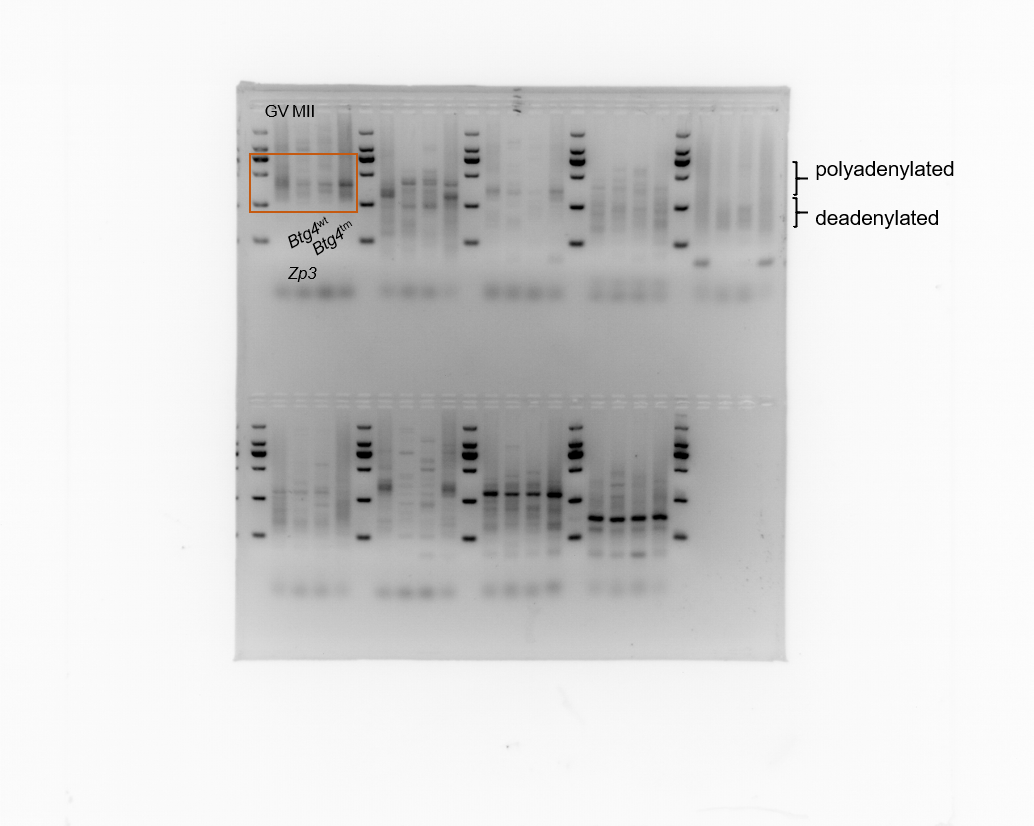

Supplement: Supplementary file 24 — Source data Fig. 4 [file 44318_2024_222_MOESM24_ESM.zip › Figure 4/4G/4G.tif]

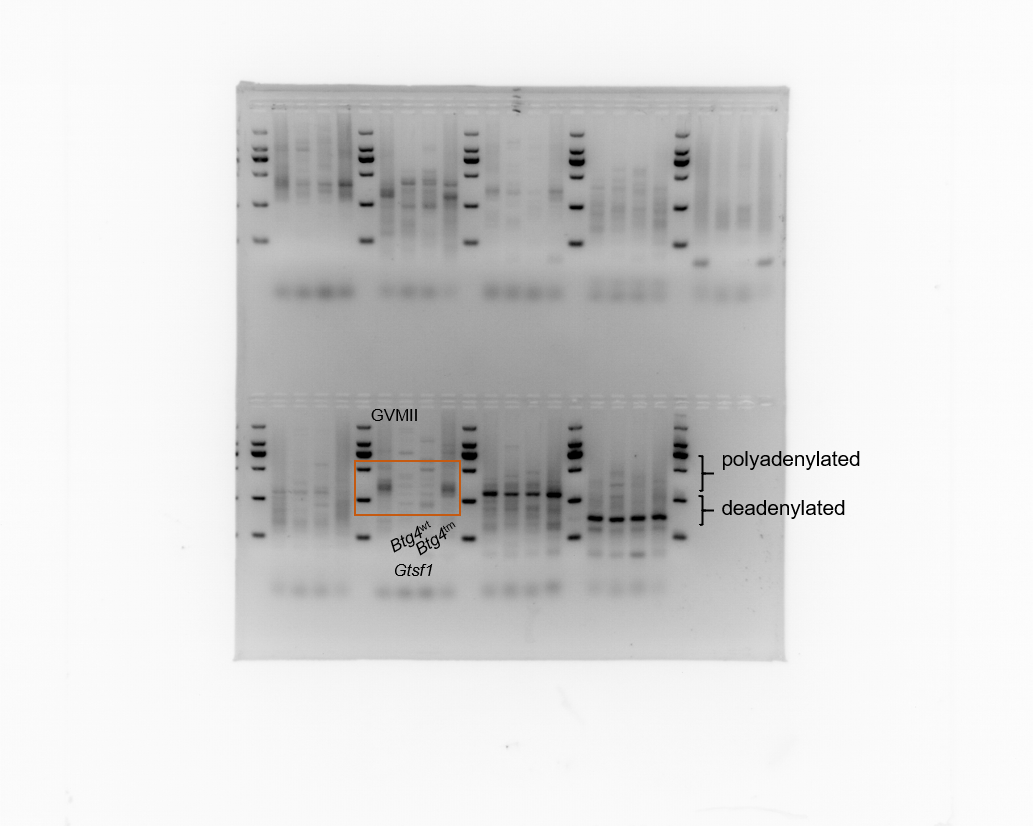

Supplement: Supplementary file 24 — Source data Fig. 4 [file 44318_2024_222_MOESM24_ESM.zip › Figure 4/4H/4H.tif]

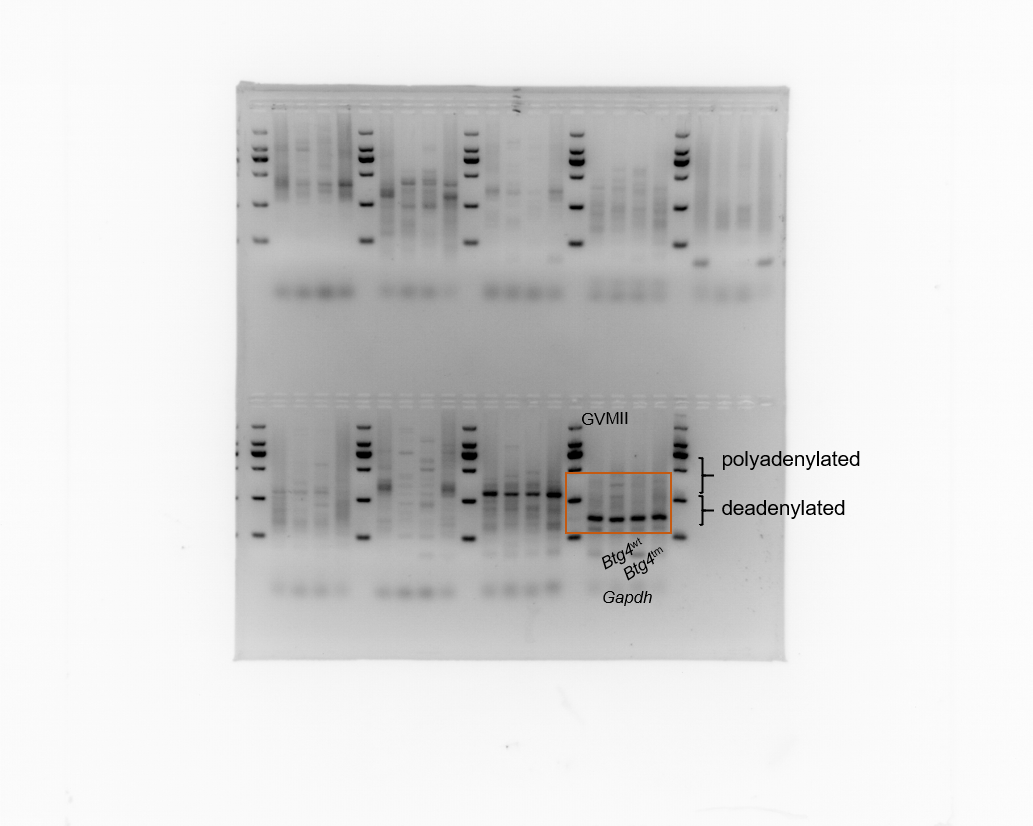

Supplement: Supplementary file 24 — Source data Fig. 4 [file 44318_2024_222_MOESM24_ESM.zip › Figure 4/4I/4I.tif]

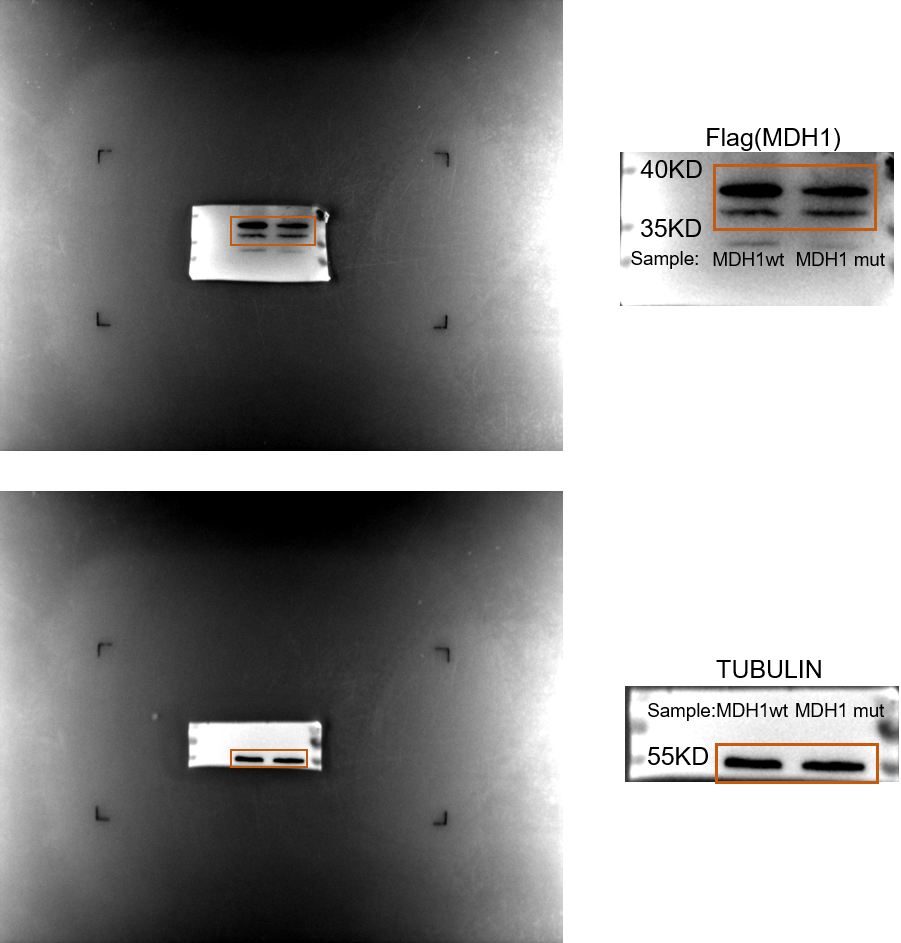

Supplement: Supplementary file 25 — Source data Fig. 5 [file 44318_2024_222_MOESM25_ESM.zip › Figure 5/5J.tif]

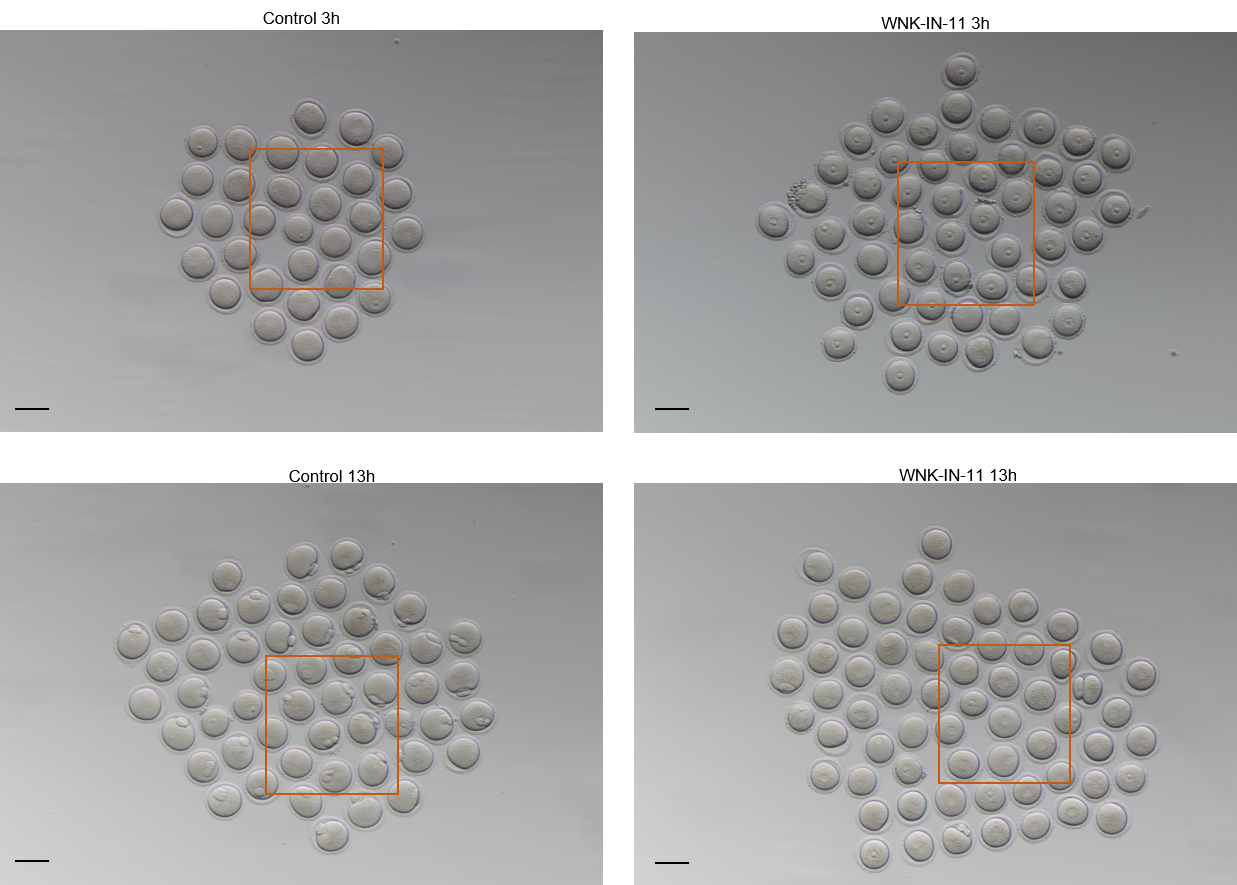

Supplement: Supplementary file 26 — Source data Fig. 6 [file 44318_2024_222_MOESM26_ESM.zip › Figure 6/6I/6I.tif]

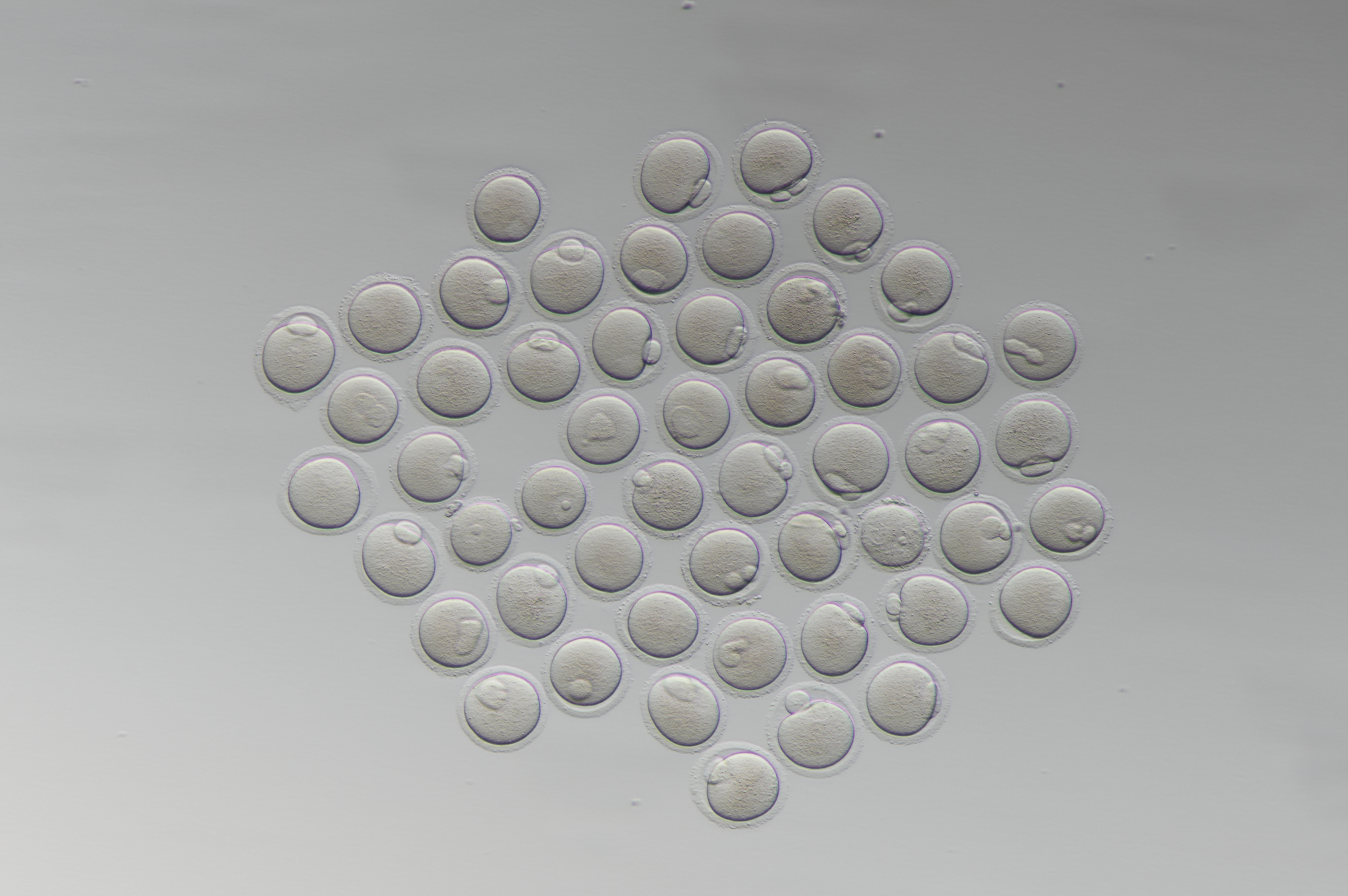

Supplement: Supplementary file 26 — Source data Fig. 6 [file 44318_2024_222_MOESM26_ESM.zip › Figure 6/6I/DMSO treatment-13h.tif]

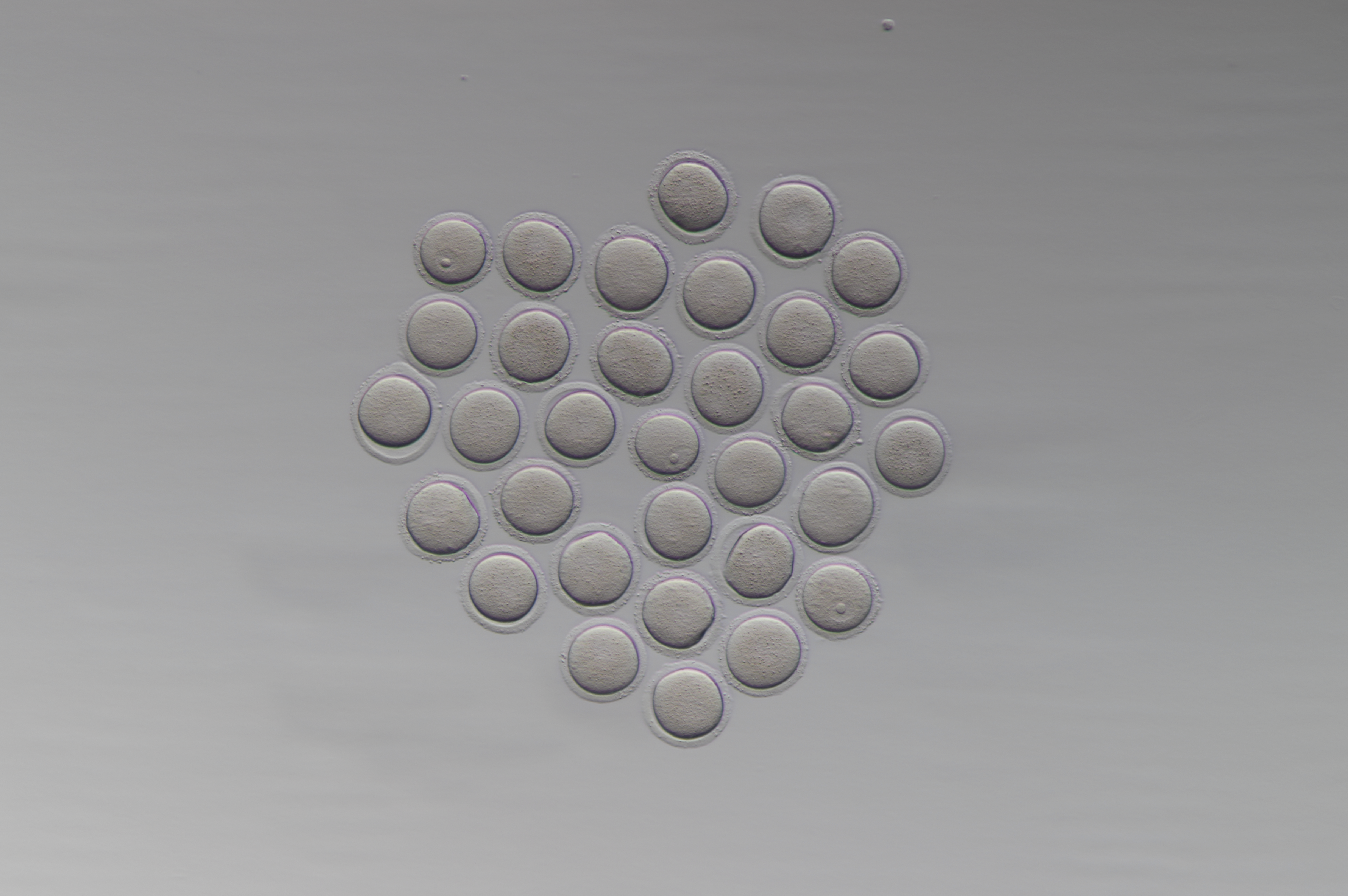

Supplement: Supplementary file 26 — Source data Fig. 6 [file 44318_2024_222_MOESM26_ESM.zip › Figure 6/6I/DMSO treatment-3h.tif]

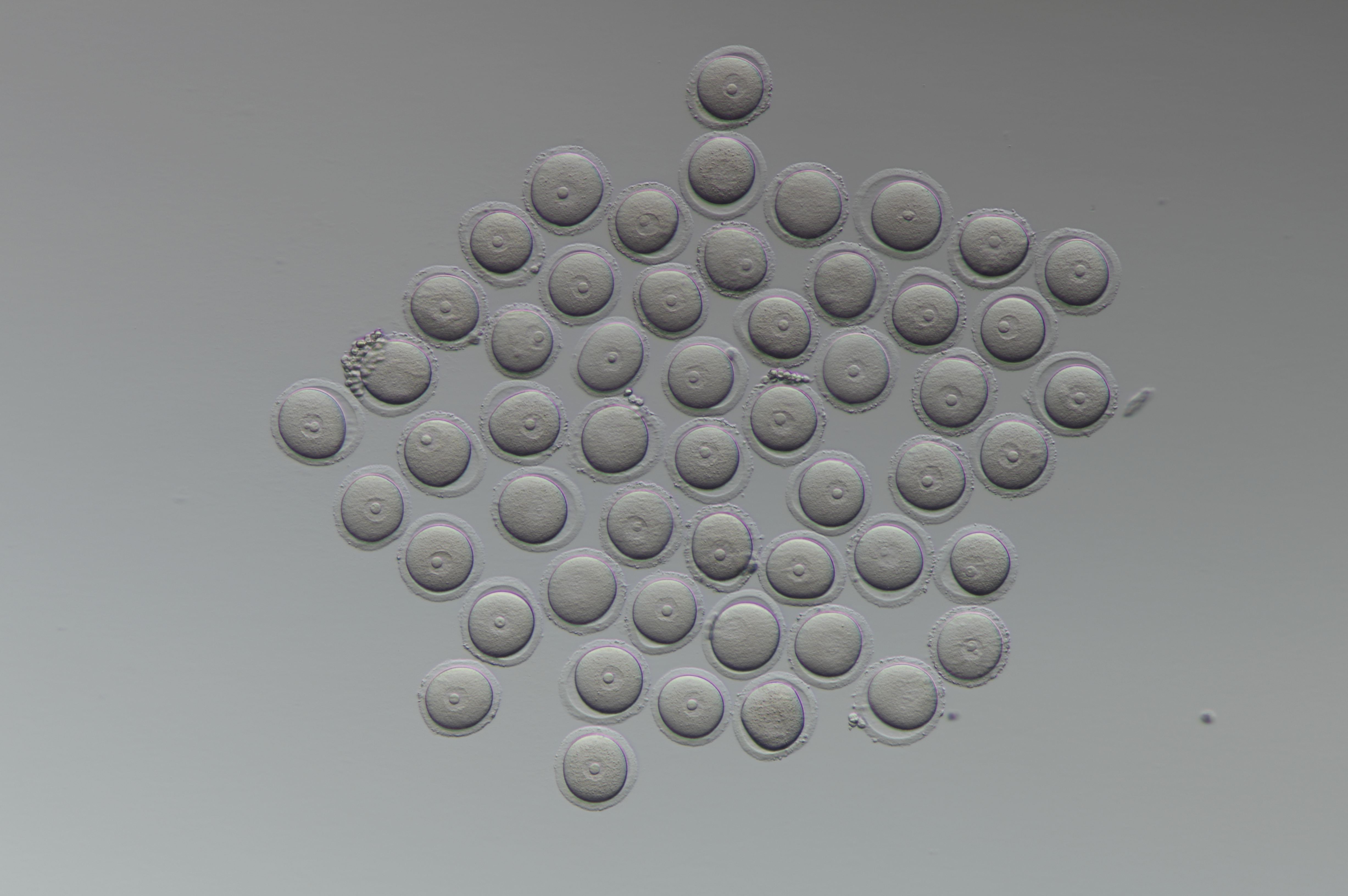

Supplement: Supplementary file 26 — Source data Fig. 6 [file 44318_2024_222_MOESM26_ESM.zip › Figure 6/6I/WNKIN11 treatment 3h.tif]

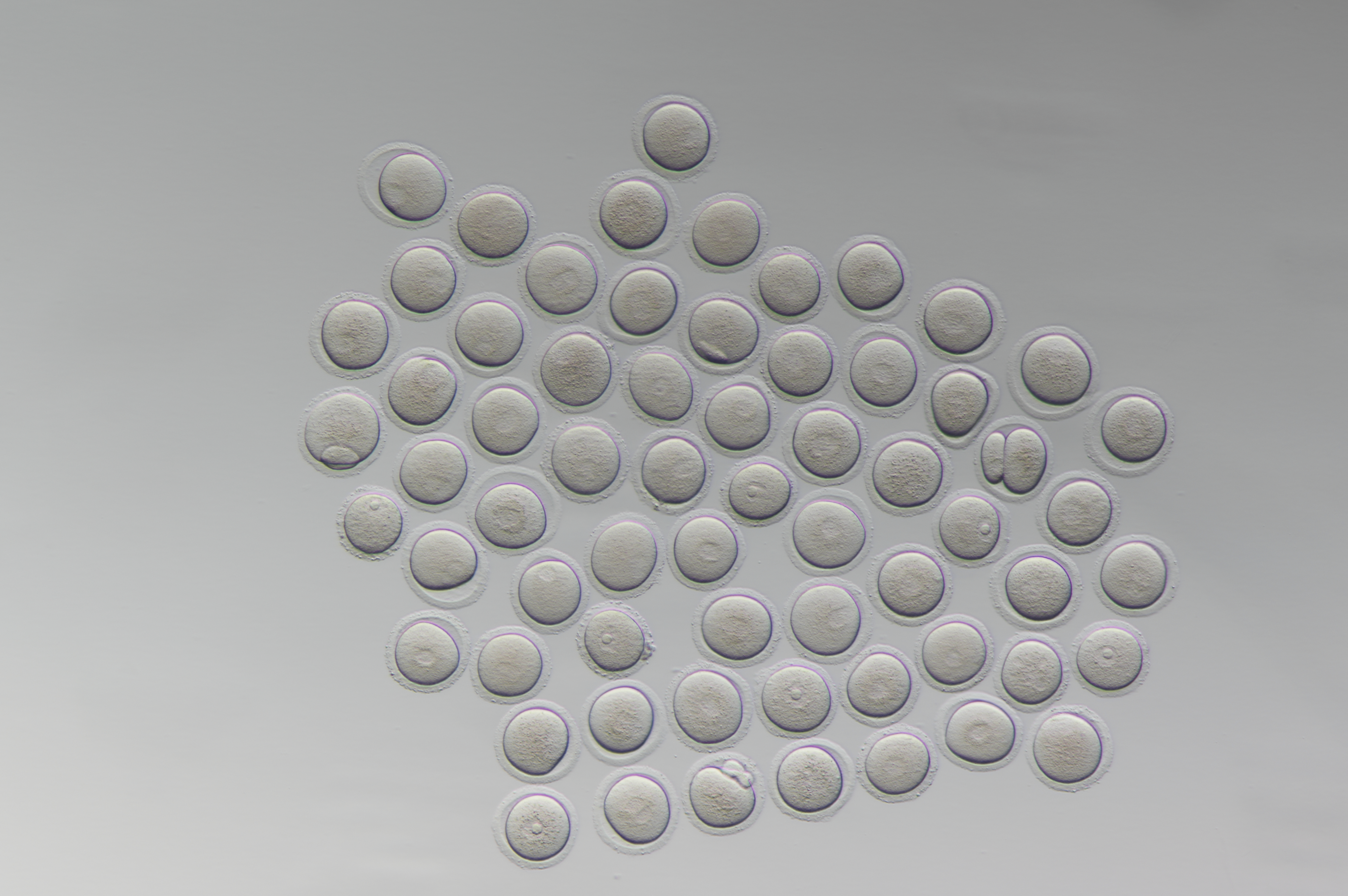

Supplement: Supplementary file 26 — Source data Fig. 6 [file 44318_2024_222_MOESM26_ESM.zip › Figure 6/6I/WNKIN11 treatment013h.tif]

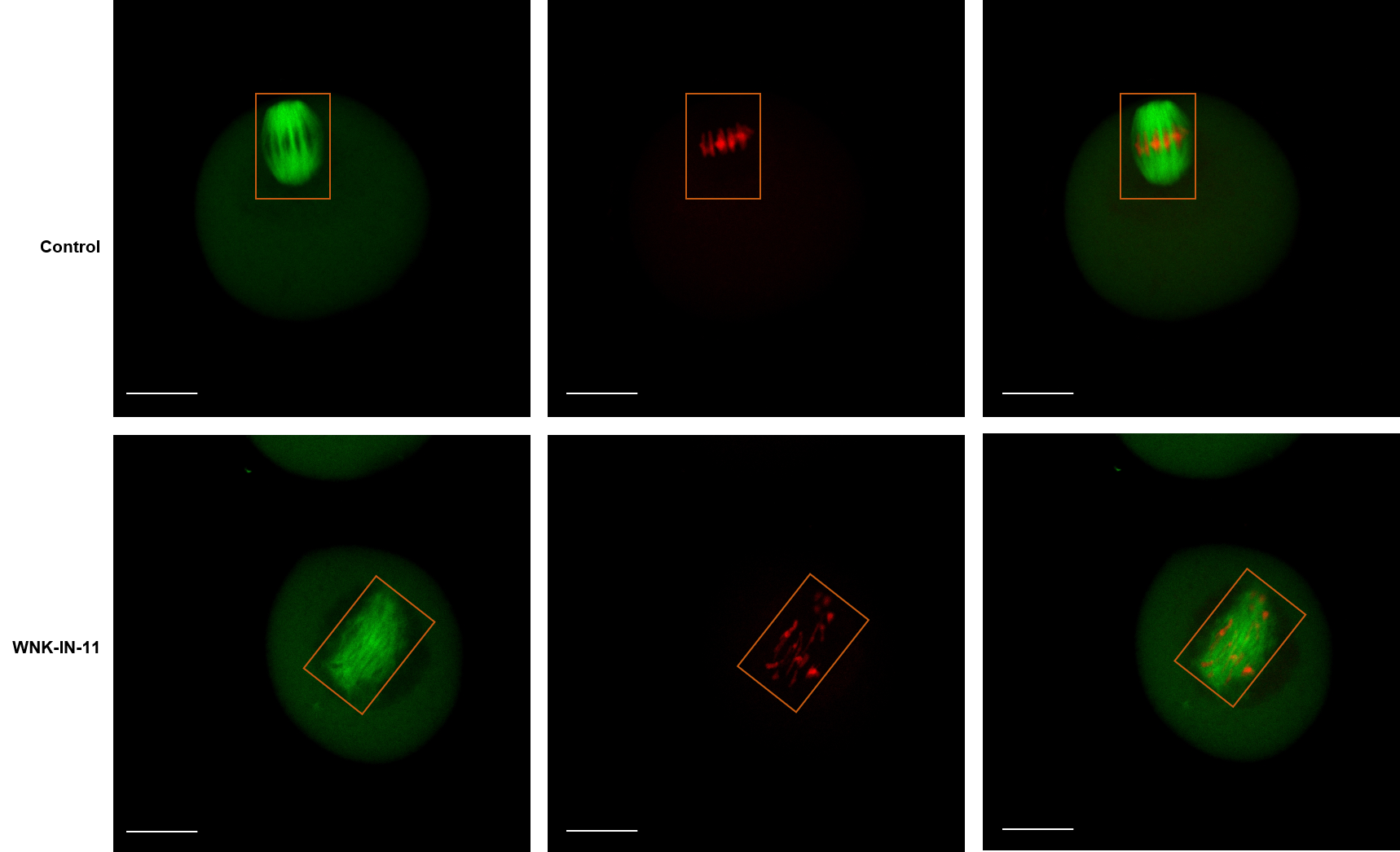

Supplement: Supplementary file 26 — Source data Fig. 6 [file 44318_2024_222_MOESM26_ESM.zip › Figure 6/6L/6L.tif]

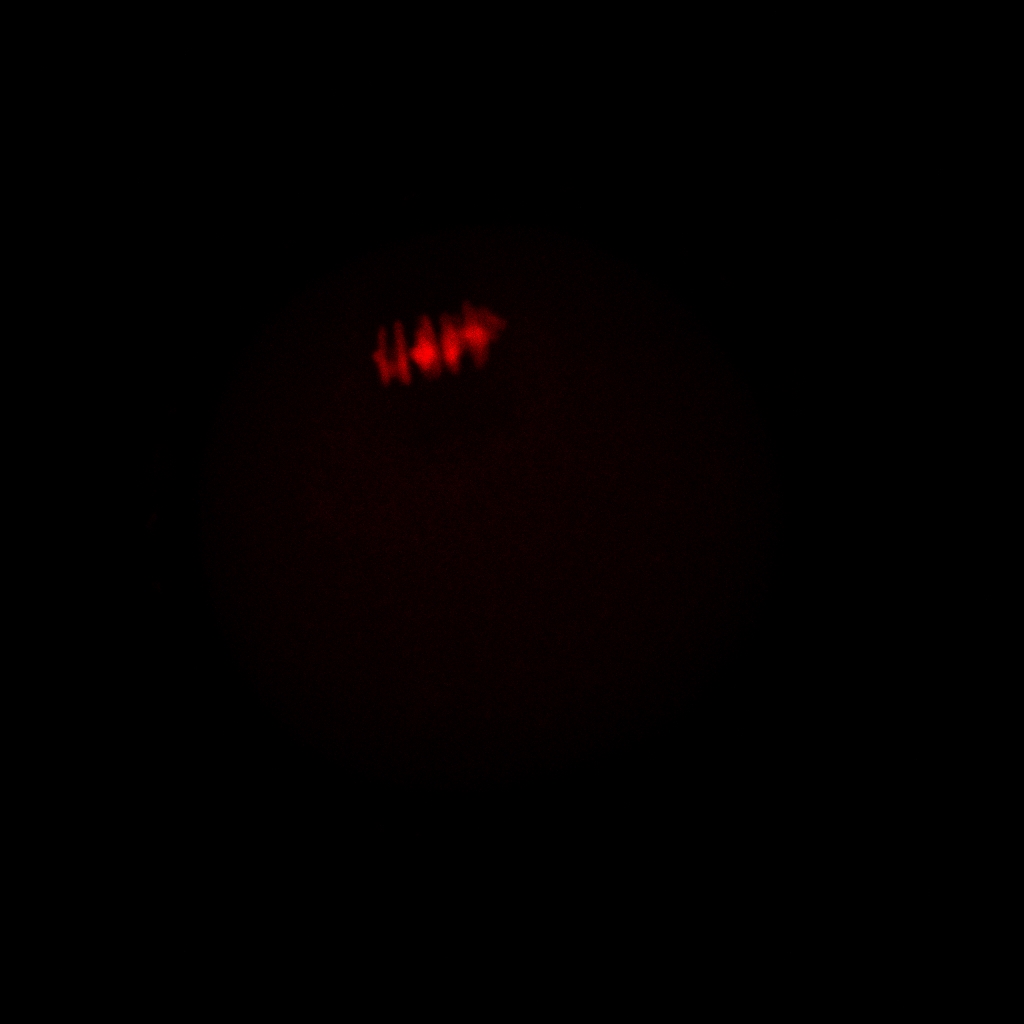

Supplement: Supplementary file 26 — Source data Fig. 6 [file 44318_2024_222_MOESM26_ESM.zip › Figure 6/6L/DMSO treatment Chromosome.jpg]

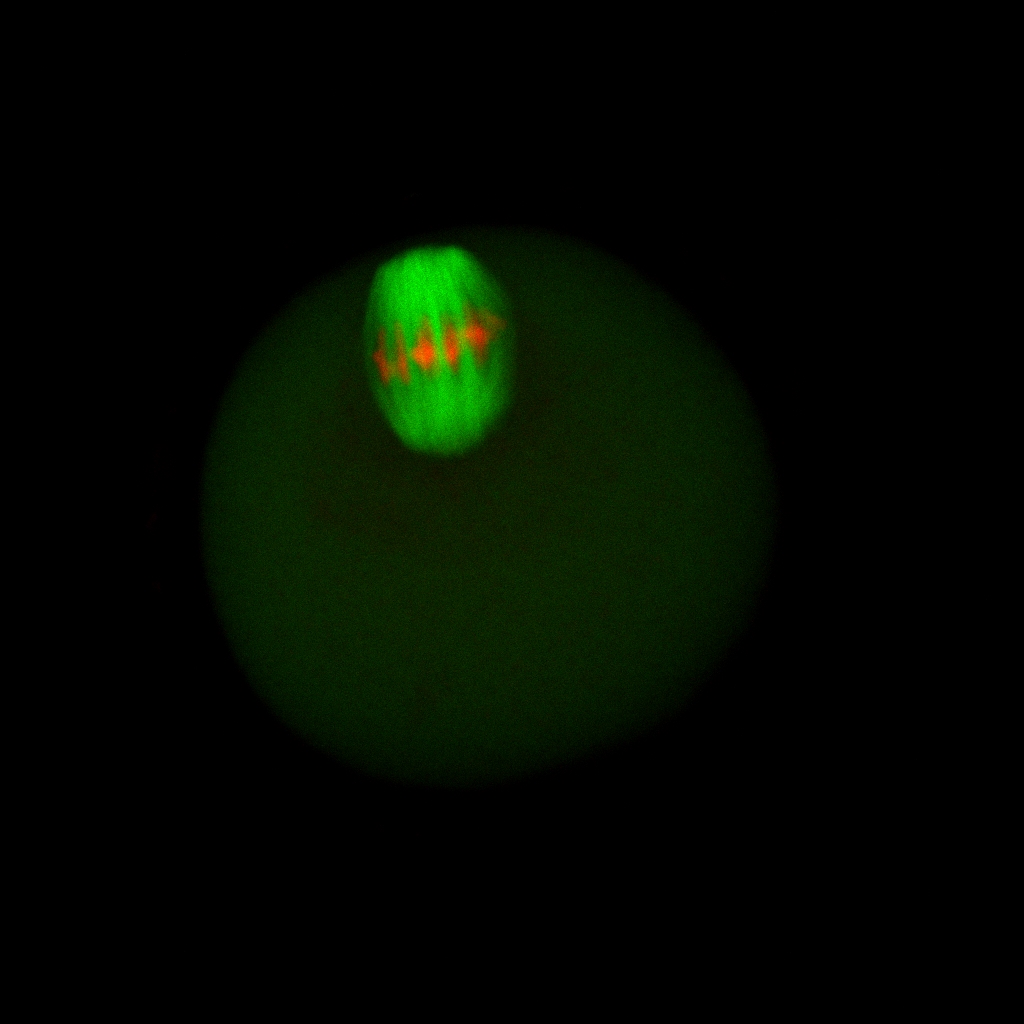

Supplement: Supplementary file 26 — Source data Fig. 6 [file 44318_2024_222_MOESM26_ESM.zip › Figure 6/6L/DMSO treatment Merge.jpg]

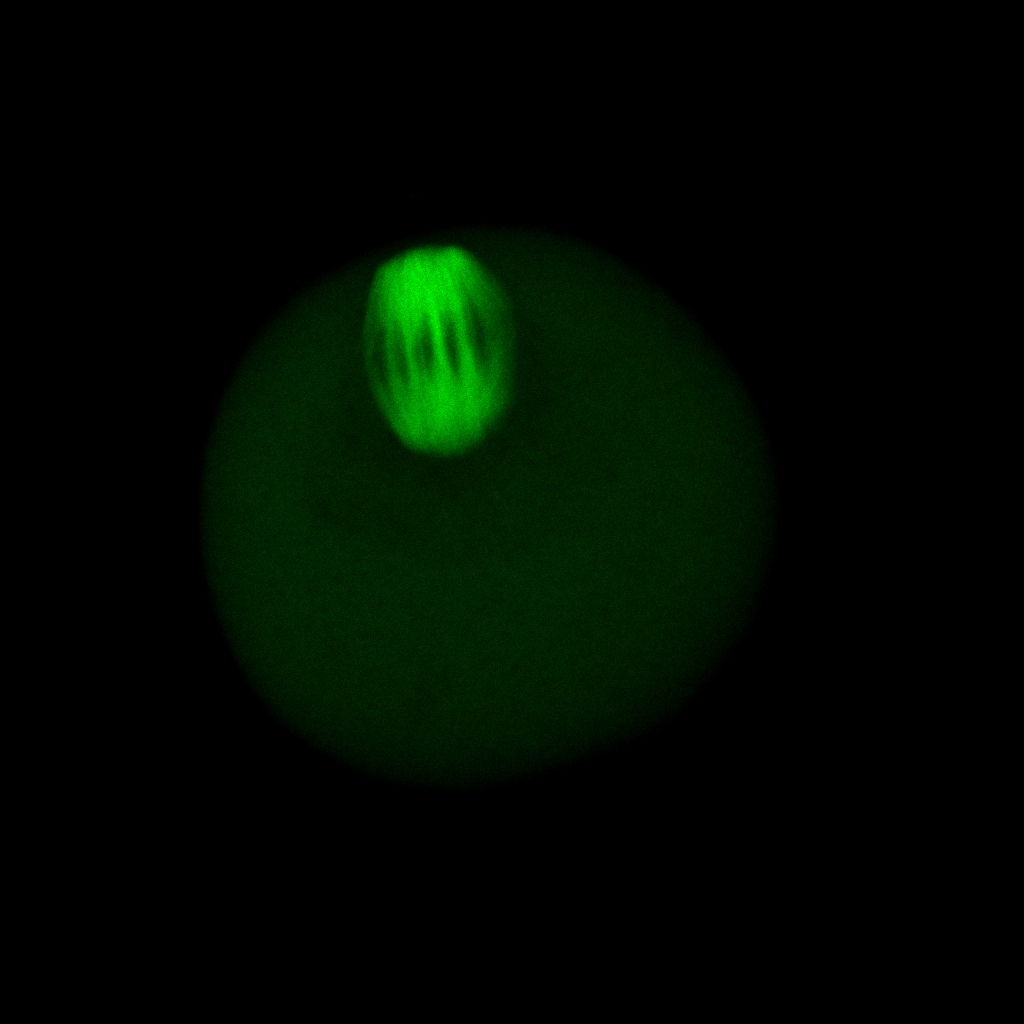

Supplement: Supplementary file 26 — Source data Fig. 6 [file 44318_2024_222_MOESM26_ESM.zip › Figure 6/6L/DMSO treatment TUBULIN.jpg]

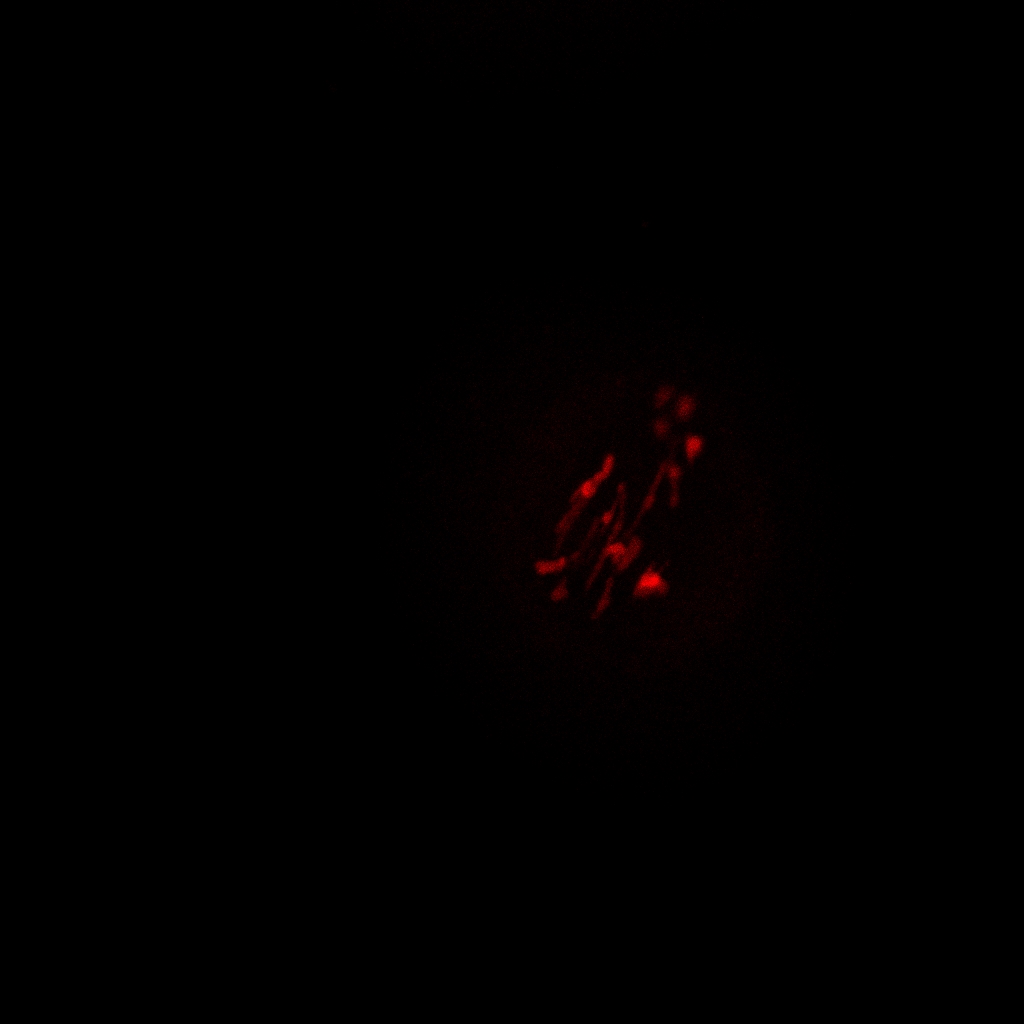

Supplement: Supplementary file 26 — Source data Fig. 6 [file 44318_2024_222_MOESM26_ESM.zip › Figure 6/6L/WNKIN11 treatment Chromosome.jpg]

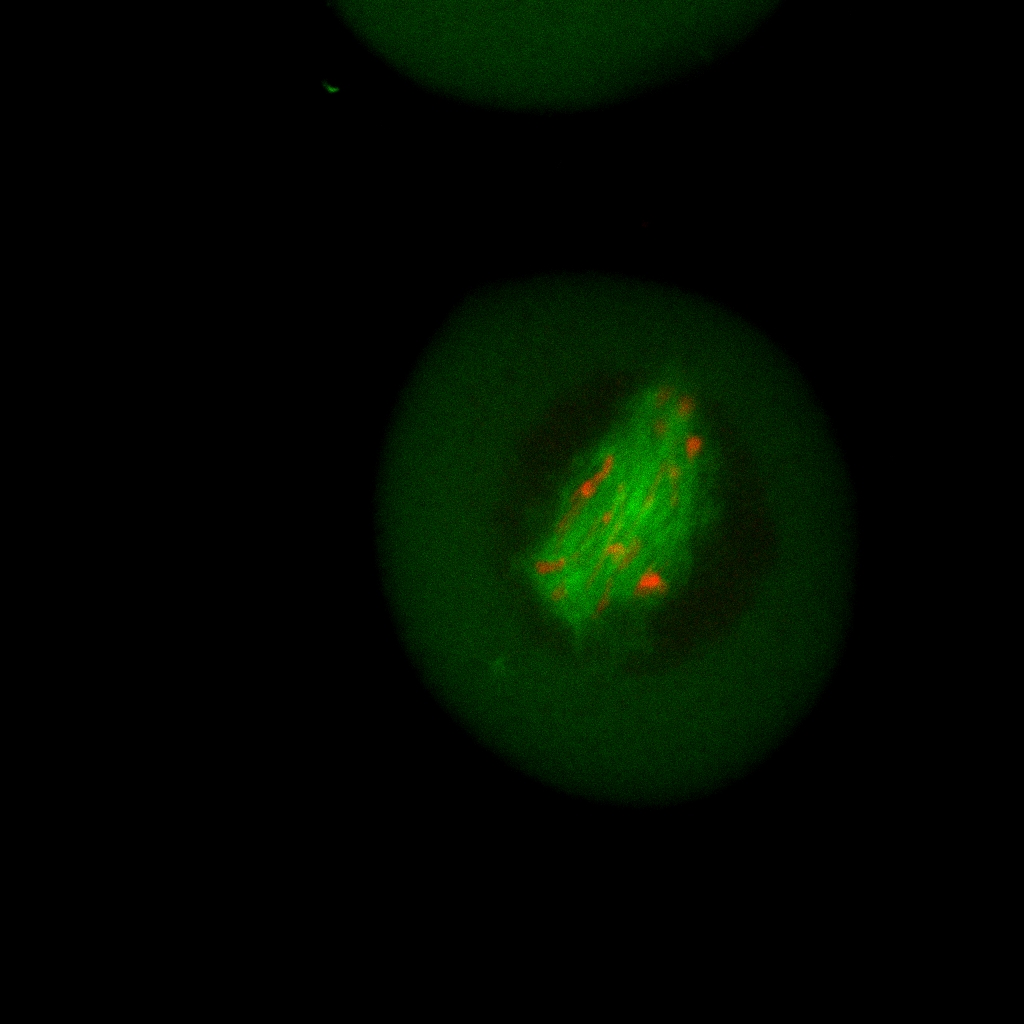

Supplement: Supplementary file 26 — Source data Fig. 6 [file 44318_2024_222_MOESM26_ESM.zip › Figure 6/6L/WNKIN11 treatment Merge.jpg]

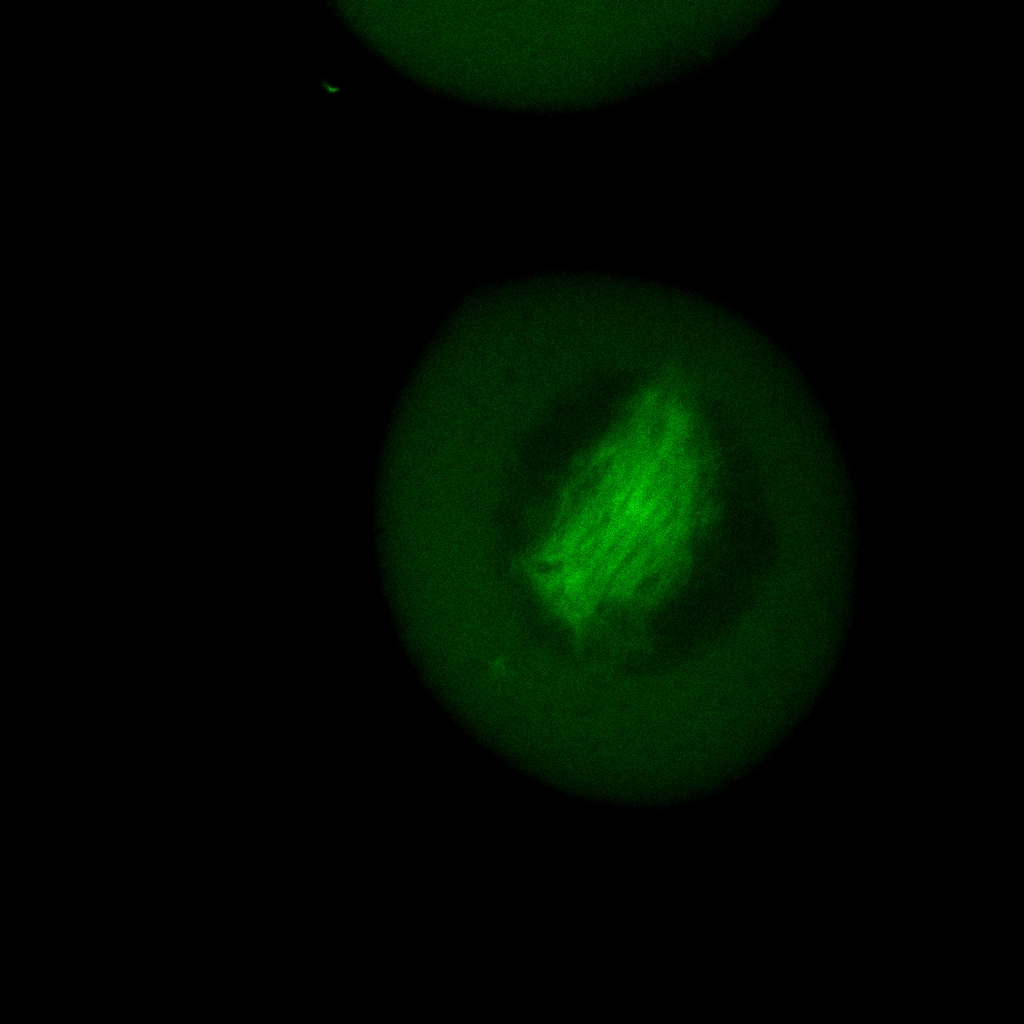

Supplement: Supplementary file 26 — Source data Fig. 6 [file 44318_2024_222_MOESM26_ESM.zip › Figure 6/6L/WNKIN11 treatment TUBULIN.jpg]
