# Supplementary material for: The global phosphorylation landscape of mouse oocytes during meiotic maturation
Source: EMBO J. 2024 Sep 10;43(20):4752–85. doi: 10.1038/s44318-024-00222-1 (PMC11480333; doi:10.1038/s44318-024-00222-1)
Supplement: Supplementary file 28 — Expanded View Figures [file 44318_2024_222_MOESM28_ESM.pdf]

Expanded View Figures

Figure EV1. Quality control for the phosphoproteomics and proteomics data. Related to Fig. 1.

(**A**) Workflow of Proteomics and Phosphoproteomics. (**B**) Principal component analysis depicting the clustering of five proteome replicates from GV, GVBD, and MII oocytes. (**C**) Heatmap illustrating the Pearson's correlation among the 15 proteome replicates obtained from GV, GVBD, and MII oocytes. (**D**) Principal component analysis depicting the clustering of five phosphoproteome replicates from GV, GVBD, and MII oocytes. (**E**) Heatmap illustrating the Pearson's correlation among the 15 phosphoproteome replicates obtained from GV, GVBD, and MII oocytes. (**F**) Coefficient of variance boxplot of proteome for each stage sample derived from GV, GVBD, and MII oocytes. Box plots: centerlines show the medians; box limits indicate the 25th and 75th percentiles; whiskers extend to the minimum and maximum. $n = 6700$. (**G**) Coefficient of variance boxplot of phosphoproteome for each stage sample derived from GV, GVBD, and MII oocytes. Box plots: centerlines show the medians; box limits indicate the 25th and 75th percentiles; whiskers extend to the minimum and maximum. $n = 8090$. (**H**, **I**) Total phosphopeptide (**G**) and peptide (**H**) intensities ranked ascending to illustrate the dynamic range of the dataset. (**J**) Bar chart showing the number of identified peptides (up), proteins (middle), and quantified proteins (bottom). (**K**) The overlap of proteins and phosphoproteins.

 © The Author(s)

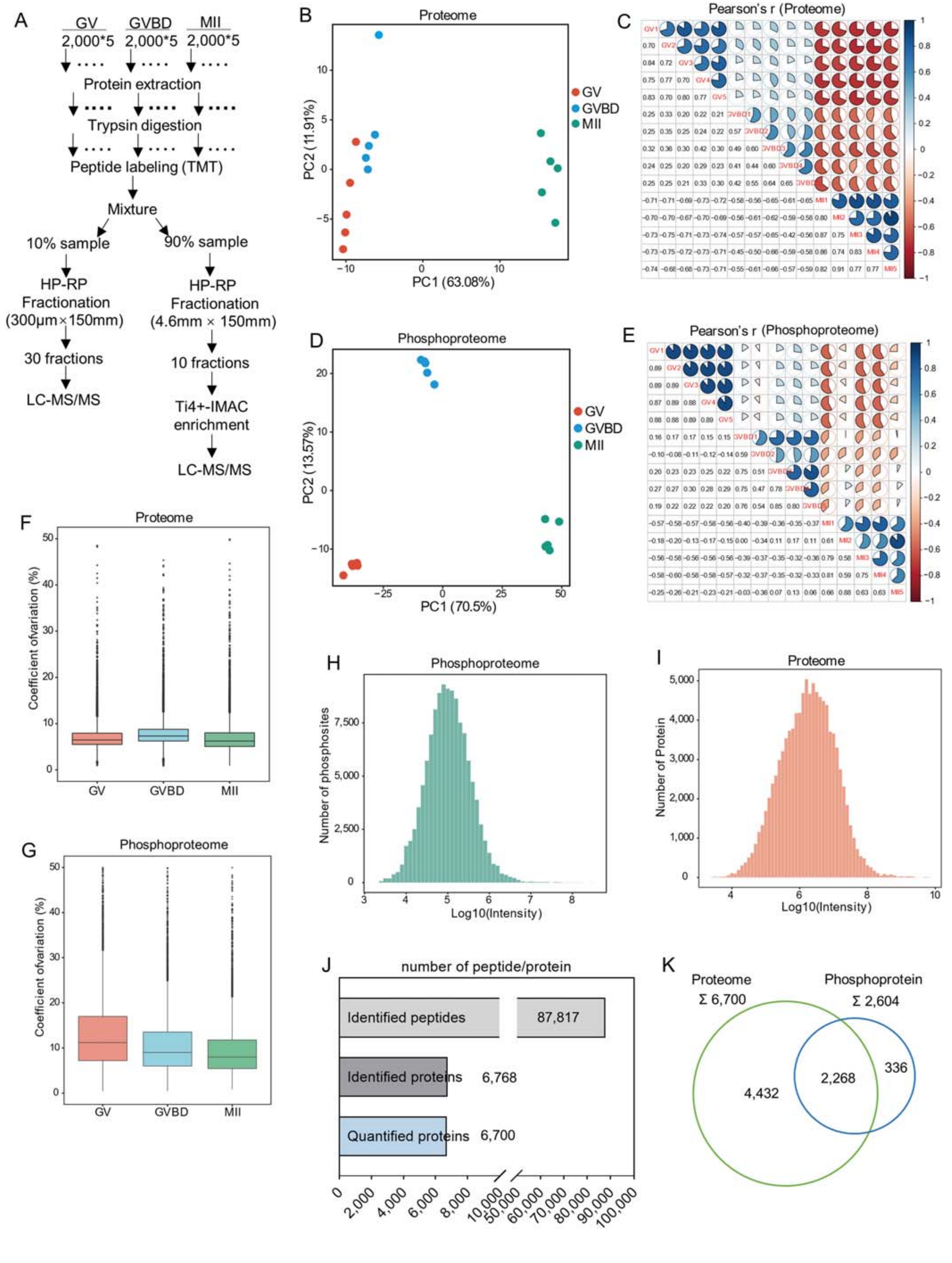
A
GV
2,000*5
GVBD
2,000*5
MII
2,000*5
Protein extraction
Trypsin digestion
Peptide labeling (TMT)
Mixture
10% sample
90% sample
HP-RP Fractionation (300μm×150mm)
HP-RP Fractionation (4.6mm × 150mm)
30 fractions
10 fractions
LC-MS/MS
Ti4+-IMAC enrichment
LC-MS/MS
B
Proteome
PC2 (11.91%)
PC1 (63.08%)
GV
GVBD
MII
C
Pearson's r (Proteome)
D
Phosphoproteome
PC2 (13.57%)
PC1 (70.5%)
E
Pearson's r (Phosphoproteome)
F
Proteome
Coefficient ofvariation (%)
GV
GVBD
MII
G
Phosphoproteome
Coefficient ofvariation (%)
H
Phosphoproteome
Number of phosphosites
Log10(Intensity)
I
Proteome
Number of Protein
Log10(Intensity)
J
number of peptide/protein
Identified peptides
87,817
Identified proteins
6,768
Quantified proteins
6,700
K
Proteome
Σ 6,700
Phosphoprotein
Σ 2,604
4,432
2,268
336

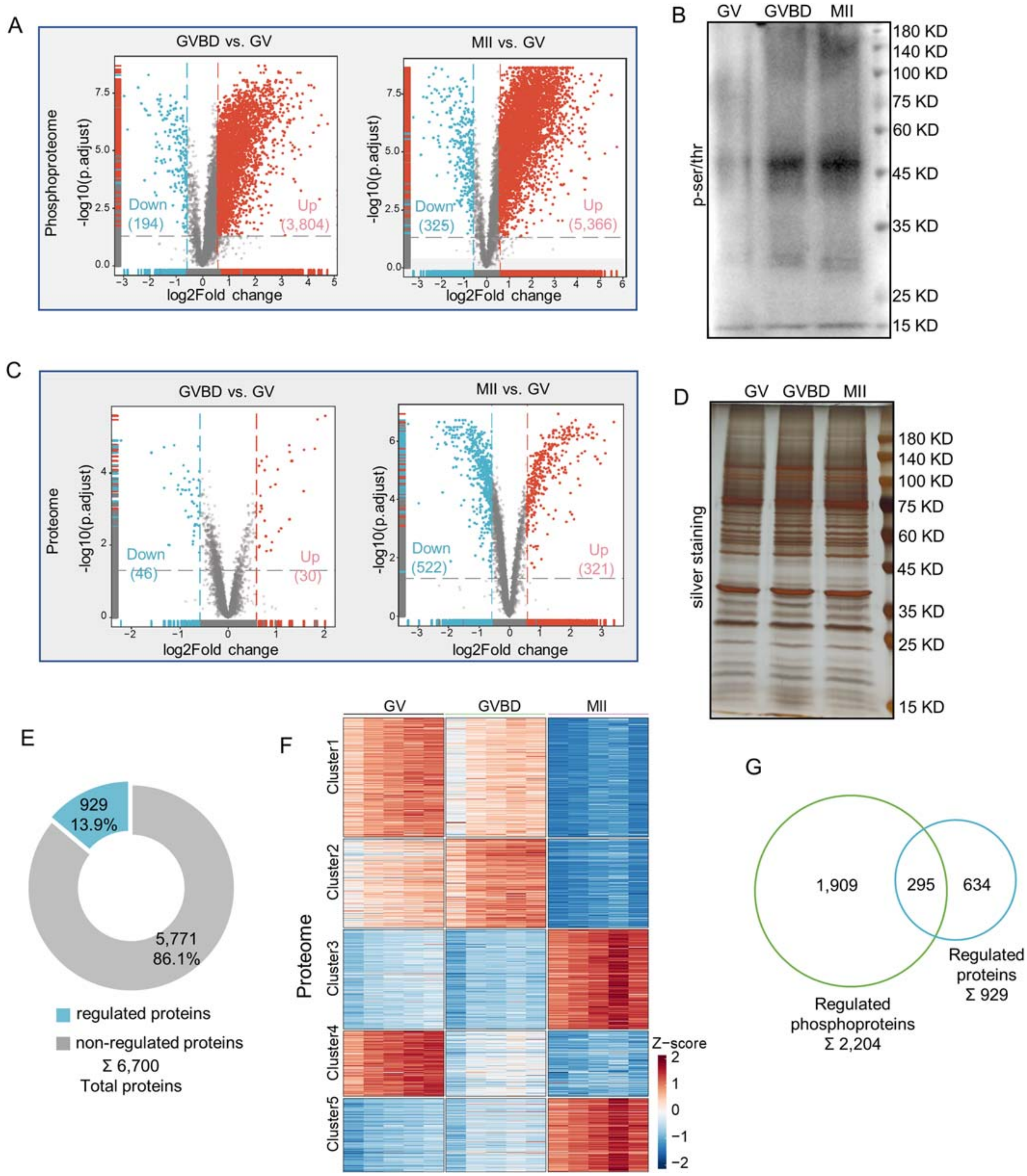
A
GVBD vs. GV
MII vs. GV
Phosphoproteome
-log10(p.adjust)
Down (194)
Up (3,804)
Down (325)
Up (5,366)
log2Fold change
B
GV GVBD MII
p-ser/thr
180 KD
140 KD
100 KD
75 KD
60 KD
45 KD
35 KD
25 KD
15 KD
C
GVBD vs. GV
MII vs. GV
Proteome
-log10(p.adjust)
Down (46)
Up (30)
Down (522)
Up (321)
log2Fold change
D
GV GVBD MII
silver staining
180 KD
140 KD
100 KD
75 KD
60 KD
45 KD
35 KD
25 KD
15 KD
E
929 13.9%
5,771 86.1%
regulated proteins
non-regulated proteins
Σ 6,700
Total proteins
F
GV GVBD MII
Proteome
Cluster1
Cluster2
Cluster3
Cluster4
Cluster5
Z−score
G
1,909
295
634
Regulated phosphoproteins
Σ 2,204
Regulated proteins
Σ 929

 © The Author(s)

Figure EV2. Features of dynamic phosphoproteome and proteome. Related to Fig. 2.

(**A**) Volcano plots showing the differentially phosphorylated proteins between GVBD and GV (left), or between MII and GV (right). Student's *t* test followed by Benjamini–Hochberg (BH) *P* value adjustment (*P*.adjust) was used for the statistical analysis. (**B**) Representative immunoblots of oocytes at GV, GVBD, and MII stages probed with pan-Ser/Thr antibody. (**C**) Volcano plots showing the differentially expressed proteins between GVBD and GV (left), or between MII and GV (right). Student's *t* test followed by Benjamini–Hochberg (BH) *P*-value adjustment (*p*.adjust) was used for the statistical analysis. (**D**) Representative silver staining of oocytes at GV, GVBD, and MII stages for protein expression detection. (**E**) Pie chart showing the number and percentage of regulated proteins. (**F**) Heatmap illustrating the dynamic changes in regulated proteins during oocyte maturation. (**G**) The overlap of regulated proteins and regulated phosphoproteins.

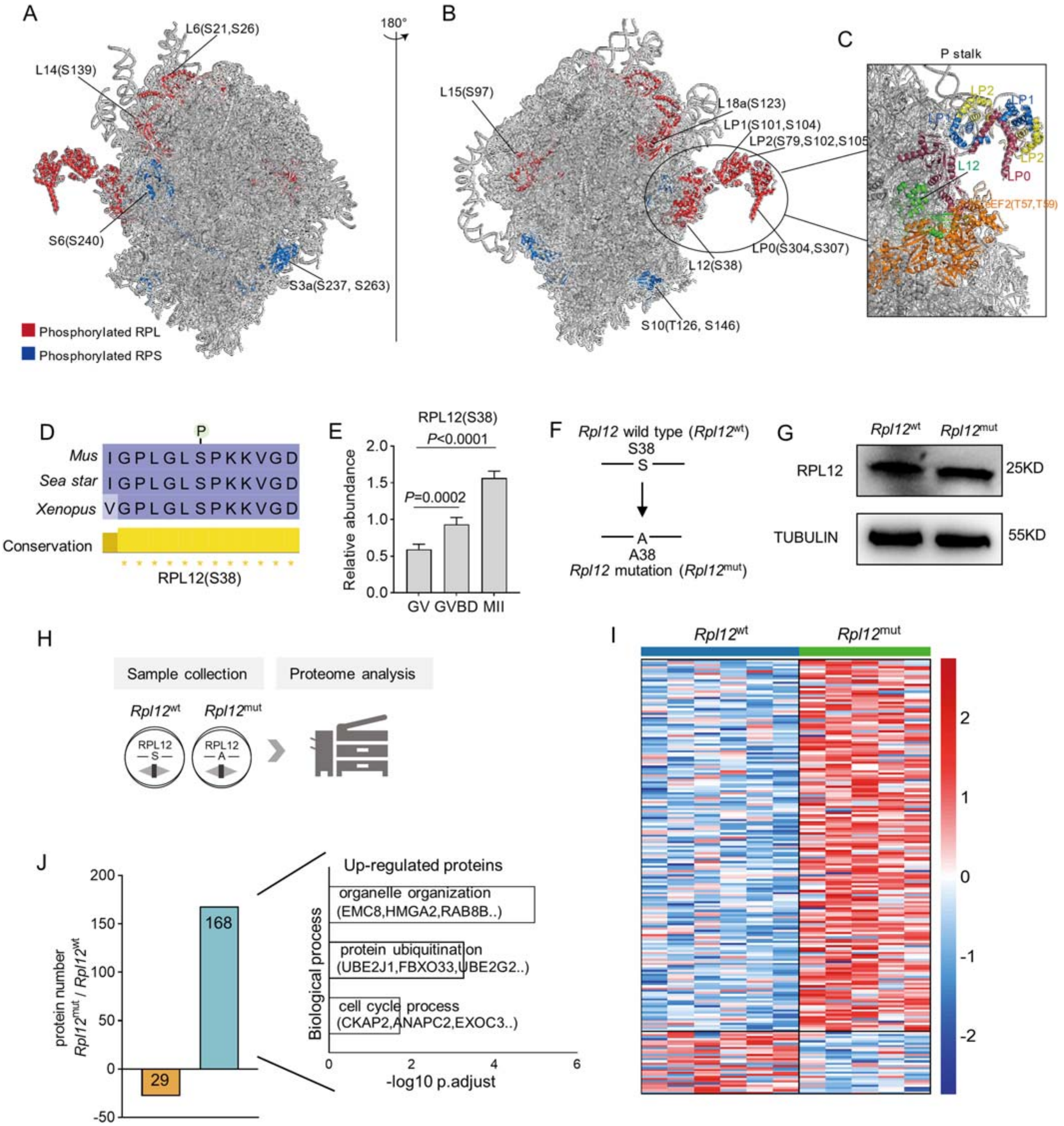

Figure EV3. RPL12-Ser38 phosphorylation participates in maintaining translational homeostasis in oocytes. Related to Fig. 5.

(**A**, **B**) Quantified phosphorylation sites of ribosome proteins (RPs) mapped to the ribosome structure (PDB: 4V6X). Phosphorylated RPSs and RPLs are shown in blue and red, respectively. RPL: Large ribosomal subunit proteins; RPS: Small ribosomal subunit proteins. (**C**) An enlarged view of the P stalk showing that RPL12-S38 is proximal to the ribosomal GTPase EEF2, RPLP0, RPLP1, and RPLP2. (**D**) Alignment and conservation analyses of RPL12 sequences flanking conserved phosphosites. (**E**) Bar chart showing the phosphorylation level of RPL12-S38 during oocyte maturation. The *p* value is labeled in the figure. Data are expressed as mean percentage ±SD from five independent replicates. Two-tailed Student's t test was used for statistical analysis, comparing to GV oocytes. (**F**) Schematic representation of the design for RPL12 phosphomutant. (**G**) Immunoblotting showing the overexpression of exogenous RPL12 ($RPL12^{wt}$ and $RPL12^{mut}$) protein in oocytes. (100 oocytes per lane). (**H**). Diagram showing the sample collection for proteomic analysis. (**I**). Heatmap showing the differentially expressed proteins between $Rpl12^{wt}$ and $Rpl12^{mut}$ oocytes. (**J**) Bar chart showing the differentially expressed proteins between $Rpl12^{wt}$ and $Rpl12^{mut}$ oocytes. The number of upregulated (up-) and downregulated (down-) proteins in $Rpl12^{mut}$ oocytes (left). Representative biological processes enriched for upregulated proteins in $Rpl12^{mut}$ oocytes (right). Benjamini–Hochberg (BH) corrected *p*-value adjustment (*p*.adjust) was used for the enrichment analyses.

 © The Author(s)

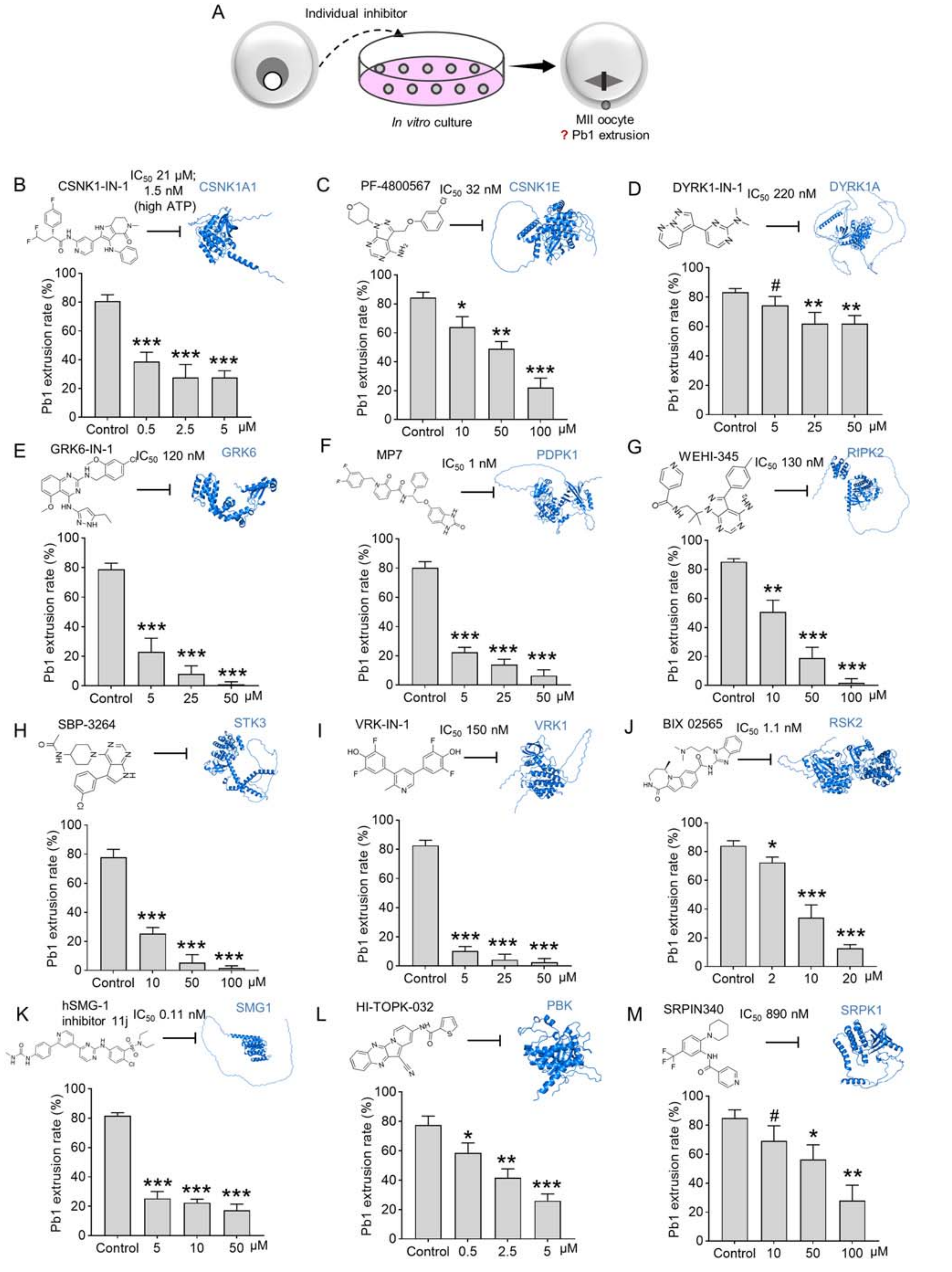
A
Individual inhibitor
In vitro culture
MII oocyte
? Pb1 extrusion
B
CSNK1-IN-1
IC50 21 μM; 1.5 nM (high ATP)
CSNK1A1
Pb1 extrusion rate (%)
Control 0.5 2.5 5 μM
***
C
PF-4800567
IC50 32 nM
CSNK1E
Control 10 50 100 μM
D
DYRK1-IN-1
IC50 220 nM
DYRK1A
Control 5 25 50 μM
#
E
GRK6-IN-1
IC50 120 nM
GRK6
Control 5 25 50 μM
F
MP7
IC50 1 nM
PDPK1
Control 5 25 50 μM
G
WEHI-345
IC50 130 nM
RIPK2
Control 10 50 100 μM
H
SBP-3264
STK3
Control 10 50 100 μM
I
VRK-IN-1
IC50 150 nM
VRK1
Control 5 25 50 μM
J
BIX 02565
IC50 1.1 nM
RSK2
Control 2 10 20 μM
K
hSMG-1 inhibitor 11j
IC50 0.11 nM
SMG1
Control 5 10 50 μM
L
HI-TOPK-032
PBK
Control 0.5 2.5 5 μM
M
SRPIN340
IC50 890 nM
SRPK1
Control 10 50 100 μM

Figure EV4. Effects of different kinase inhibitors on oocyte maturation. Related to Fig. 6.

(**A**) Schematic presentation of the inhibitor treatment experiment. (**B–M**) Quantitative analysis of the Pb1 extrusion rate in oocytes treated with different inhibitors. Data are expressed as mean percentage ±SD from three independent replicates in which at least 100 oocytes were analyzed for each group. Two-tailed Student's *t* test was used for statistical analysis, comparing to control group (DMSO treatment). $^{\#}P > 0.05$, $^{*}P < 0.05$, $^{**}P < 0.01$, $^{***}P < 0.001$.

 © The Author(s)

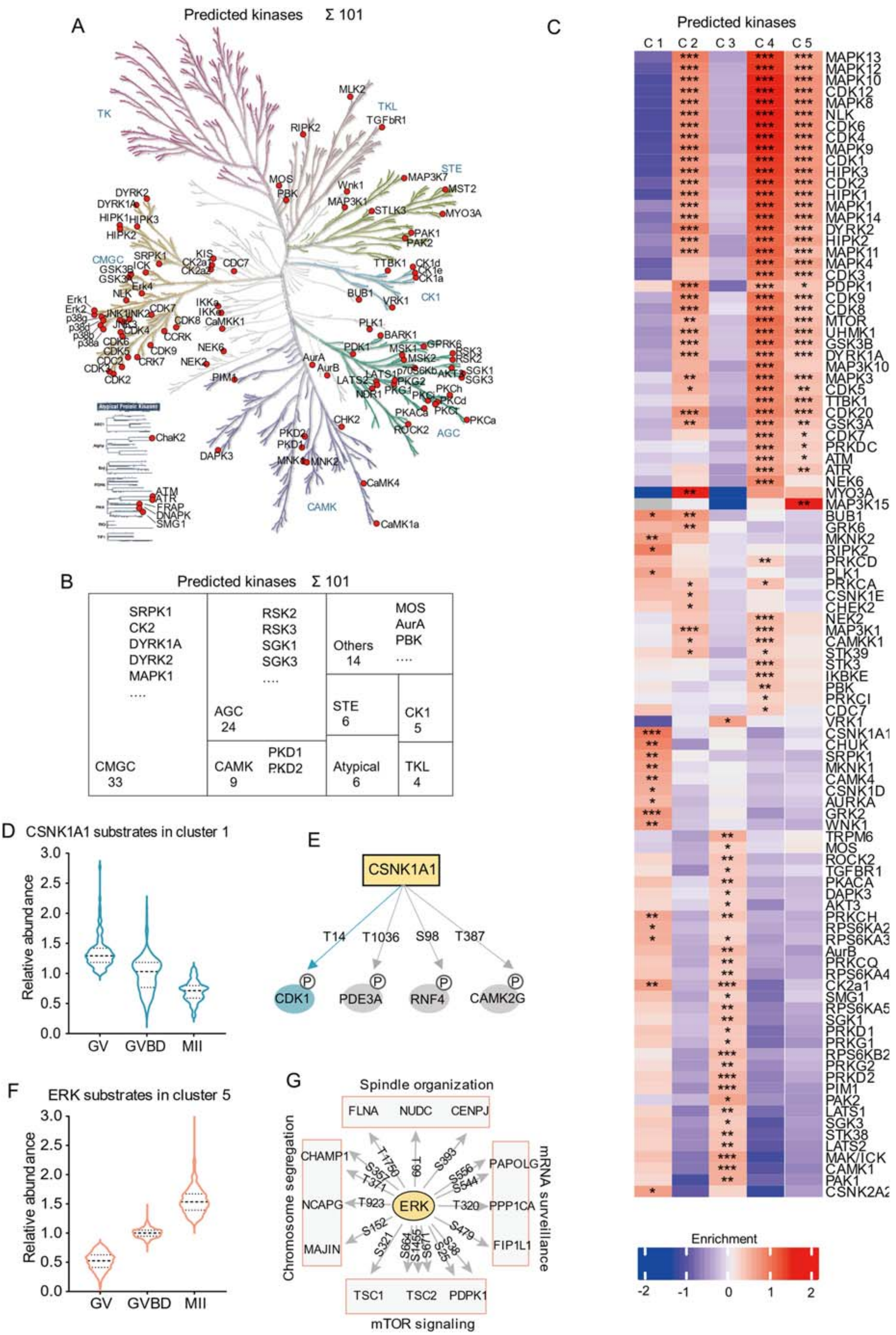
A
Predicted kinases Σ 101
TK
TKL
STE
CMGC
CK1
AGC
CAMK
B
Predicted kinases Σ 101
SRPK1 CK2 DYRK1A DYRK2 MAPK1 ....
CMGC 33
RSK2 RSK3 SGK1 SGK3 ....
AGC 24
MOS AurA PBK ....
Others 14
STE 6
CK1 5
CAMK 9
PKD1 PKD2
Atypical 6
TKL 4
C
Predicted kinases
C 1 C 2 C 3 C 4 C 5
Enrichment
-2 -1 0 1 2
D CSNK1A1 substrates in cluster 1
Relative abundance
GV GVBD MII
E
CSNK1A1
T14 T1036 S98 T387
CDK1 PDE3A RNF4 CAMK2G
F ERK substrates in cluster 5
Relative abundance
GV GVBD MII
G
Spindle organization
FLNA NUDC CENPJ
Chromosome segregation
CHAMP1 NCAPG MAJIN
mRNA surveillance
PAPOLG PPP1CA FIP1L1
ERK
TSC1 TSC2 PDPK1
mTOR signaling

Figure EV5. Enrichment analysis of the predicted kinases in oocytes. Related to Fig. 6.

(**A**) Kinase tree showing the predicted kinases annotated to the major kinase families. (**B**) The number of predicted kinases in each kinase family. (**C**) Heatmap showing the predicted kinases enriched in each cluster. $^{*}P < 0.05$, $^{**}P < 0.01$, $^{***}P < 0.001$. Benjamini–Hochberg (BH) corrected *P* value adjustment (*P*.adjust) was used for the enrichment analyses. (**D**) Phosphorylation levels of the predicted CSNK1A substrates in cluster 1 during oocyte maturation. $n = 191$. (**E**). Networks showing the representative phosphosites in annotated substrates (KSPN) of CSNK1A1 kinase. Proteins are shown as nodes and phospho-residue is indicated by the number. (**F**) Phosphorylation levels of the predicted ERK substrates in cluster 5 during oocyte maturation. $n = 672$. (**G**) Networks showing the representative phosphosites in annotated substrates (KSPN) of ERK kinases. Proteins are shown as nodes and phospho-residue is indicated by the number.

 © The Author(s)
